# Recent Overview of Potent Antioxidant Activity of Coordination Compounds

**DOI:** 10.3390/antiox12020213

**Published:** 2023-01-17

**Authors:** Hany M. Abd El-Lateef, Tarek El-Dabea, Mai M. Khalaf, Ahmed M. Abu-Dief

**Affiliations:** 1 Department of Chemistry, College of Science, King Faisal University, Al-Ahsa P.O. Box 400, Saudi Arabia; 2 Chemistry Department, Faculty of Science, Sohag University, Sohag 82534, Egypt; 3 Chemistry Department, Faculty of Science, King Salman International University, Ras Sudr, Sinai 46612, Egypt; 4 Chemistry Department, College of Science, Taibah University, Madinah P.O. Box 344, Saudi Arabia

**Keywords:** chelation, modification, EPR, antioxidant performance, food protection, dietary supplements, high-quality

## Abstract

During recent decades, the complexation of organic ligands toward several metal ions of s-p and d-block has been applied as a plan to enhance its antioxidant performance. Due to their wide range of beneficial impacts, coordination compounds are widely used in industries, specifically in the medicinal and pharmaceutical fields. The activity is generally improved by chelation consequently knowing that the characteristics of both ligands and metals can lead to the development of greatly active compounds. Chelation compounds are a substitute for using the traditional synthetic antioxidants, because metal chelates present benefits, including a variety in geometry, oxidation states, and coordination number, that assist and favor the redox methods associated with antioxidant action. As well as understanding the best studied anti-oxidative assets of these compounds, coordination compounds are involved in the free radical scavenging process and protecting human organisms from the opposing effects of these radicals. The antioxidant ability can be assessed by various interrelated systems. The methodological modification offers the most knowledge on the antioxidant property of metal chelates. Colorimetric techniques are the most used, though electron paramagnetic resonance (EPR) is an alternative for metallic compounds, since color does not affect the results. Information about systems, with their benefits, and restrictions, permits a dependable valuation of the antioxidant performance of coordination compounds, as well as assisting application in various states wherever antioxidant drugs are required, such as in food protection, appropriate good-packaged foods, dietary supplements, and others. Because of the new exhaustive analysis of organic ligands, it has become a separate field of research in chemistry. The present investigation will be respected for providing a foundation for the antioxidant properties of organic ligands, future tests on organic ligands, and building high-quality antioxidative compounds.

## 1. Introduction

Human life depends on our body’s metabolic processes, equilibrium, and optimally performing repair systems. Free radicals are chemical entities that are reactive and have one or more unpaired electrons. Free oxygen radicals play a vital part in our bodies normal functioning [1,2,3]. In vivo, these have been developed as part of metabolism and have little sensitivity, and are capable of attacking DNA, proteins and fatty acids [4], which can contribute to oxidative stress, and thus is the cause of many human physical abnormalities. On the other hand, O_2_ possesses a dual face; it serves as a cause of reacting O_2_/N_2_ species (ROS/RNS) that in excesses can cause major health problems [5,6,7,8], as well as central nervous system neurodegeneration, such as Alzheimer’s disease [9] and aging [10,11,12]. It is commonly understood that oxygen is required for the process of breathing and that its absence is associated with death. All of our biological processes are guided by proper oxygen oxidation [13,14]. (ROS/RNS) species are defined chemically as particles containing at least one unpaired electron [15]. Antioxidants are chemical substances that, while found in low doses compared to that of an oxygen electrode, can delay or prevent oxidation [16]. Antioxidants are the body’s natural first line of defense against dangerous chemicals identified as free radicals, and they must respond quickly to free radicals to stop biomolecules from getting damaged. With greater exposure to free radicals, the requirement for antioxidants becomes even more crucial. Free radical exposure can be increased by pollution, cigarette smoke, medications, sickness, stress, and even exercise [17]. Body cells manufacture them in reaction to free radicals [18,19,20,21]. Antioxidants are commonly utilized as catalysts in antibiotics for anti-inflammatory, antifungal, antibacterial, and antiviral purposes, as well as in the industry for anticorrosion [22]. Organic compounds are a subclass of ligands that contain a variety of donor orbitals and exhibit attractive coordination configurations with metals [23]. Organic compounds were frequently employed as ligands due to their high stability and solubility in common solvents such as EtOH, MeOH, CHCl_3_, and DMF. The type of functional group linked with aromatic organic rings determines their biological activity. Organic molecules, as well as their metallic complexes, have been increasingly important in recent years due to their exceptional living uses [24,25]; anticancer [26,27], antibacterial [28], antitumor [29], antifertility and antifungal [30], antioxidant [29], herbicidal [31,32], and ant-proliferative [33] properties are among the intrinsic biological actions of these substances. Furthermore, organic ligands have photoluminescence [34], fluorescence [35], potentiometric action caring [36], anthelmintic [37], and aggregation [38] capabilities. Aromatic compounds are simple to synthesize and can bind to a wide range of metal ions through various oxidation states and symmetries [39,40]. Their complexes are recognized as including some of the most vital stereo-chemical modeling techniques throughout the main group and transition metal-ligand chemistry [41] because of their preparative availability and physical variety. However, the binding of metal ions with these compounds seems to have a huge spectrum of applications in analytical chemistry, the food industry, the dye industry, catalysis, fungicidal, agrochemical, and biological activities, as well as a slight decrease in the cytotoxicity from both ions and Aryl ring [42,43,44,45,46]. The investigation of metal-derived antioxidants has garnered considerable care and power in an attempt to generate compounds with great potential for scavenging free radicals linked with several ailments and diseases induced through ROS. Synthetic antioxidants are now commonly employed since they are more effective and less expensive than natural antioxidants. Several Schiff-base metal chelates are currently being studied as potent ROS scavengers and antioxidants [47,48]. Until now, no extensive reports on the antioxidant abilities of Schiff bases, and their potential metallic chelates, have been published. According to the results, it is necessary to investigate new antioxidants that can work inside an organism’s defense based on their chemical structure and unique replacement patterns that include both ROS deactivation and suppression of their formation.

## 2. Free Radicals & Antioxidants

Radicals are reactive chemical units that are reactive and also have one or more unpaired electrons. In vitro tests have been generated as a portion of cellular metabolism and have no specificity, attacking DNA, peptides, and fatty acids [49], as well as being linked to diseases such as cancer and vascular disease [50,51], and pathological processes of the nervous system including Alzheimer’s disease [49,52] and growing older [53]. Antioxidants are organic molecules that, once supplied for low quantities associated with those found in an oxygen electrode substrate, can retard or prevent degradation [54]. In contrast to an antioxidant, which protects the neutral substrate from oxidation, a radical is a reduced living species. Antioxidants react by inhibiting or avoiding other compounds from decaying. The early studies on the function of antioxidants in genetics concentrated on their effectiveness in reducing oxidative degradation of cellular membranes [55,56,57]. However, the findings of vitamins E, C and A [58], as well as the explanation of the mechanism for oxidative stress prevention by vitamin E [59], were watershed moments in comprehending the role of antioxidants in living beings. Antioxidants are typically divided into two types: non-enzymatic and enzymatic. They include numerous compounds with various sites of action and modes, as well as various final effects. This variety provides each of them with particular role functions within the body. It must be noted that the system for cooperating antioxidative enzymes, such as glutathione reductase (GRd), superoxide dismutase enzymes (SODs), glutathione peroxidase (GPx) and catalase (CAT), provides the most effective antioxidant defense [60]. Low-molecular-weight antioxidants, such as coenzyme Q, vitamin C, E, carotenoids, microelements, and glutamine, are also involved in aggressive radical inactivation. Some of them are produced by the body, such as ubiquinone, glutamate, albumin, metallothioneins, and uric acid [61], but the majority are external compounds that come from nature, including plants (carotenoids, flavonoids, coumarins, stilbenes, phenolic acids, lignans, vitamins, and organosulfur molecules) and minerals (Se, Mn, and Zn). As endogenous antioxidants engaged in free radical protection are unable to shield the body from ROS, exogenous antioxidants are required. Dietary supplements can also be used to supply antioxidants to the body. Synthetic antioxidants are bioequivalent to natural antioxidants; for example, bio vitamin C vs. chemically manufactured L-ascorbic acid, or synthetic and natural R, R-α-tocopherol. Antioxidants are also utilized as additives in the food, cosmetic, and pharmaceutical industries to keep unstable compounds from oxidizing. This mainly applies to phenolic-structured synthetic antioxidants used in food, such as BHT [butylated hydrotoluene], BHA [butylated hydroanisole], and TBHQ [tert-butylated hydroquinone] [62]. Antioxidants vary in their capacity to neutralize free radicals. It has been demonstrated that antioxidant potential is highly associated with the number of powerful groups, such as NH_2_, OH, and the position of each group in the series to meta < para < ortho, starting with the most active and progressing toward the least active [63]. It emphasized that antioxidants can perform a variety of processes, not only scavenging radicals but also separating metal ions decaying H_2_O_2_ or hydroperoxides, softening aggressive pro-oxidants, and boosting endogenous antioxidant effects, such as fixing the resulting cellular destruction. Therefore, antioxidants are often categorized as principal or sequence-flouting antioxidants and intermediate or preventing antioxidants [64]. Main antioxidants effectually stop the oxidation process through sequestering ROS/RNS, whereas 2nd antioxidants operate implicitly via coordination through metal (iron) ions [65,66] as well as extra detailed activities such as the initiation of protective parameters, anti-inflammatory suppression of NADPH oxidase [nicotinamide adenine dinucleotide phosphate oxidase], inhibition of xanthine oxidase, and regulation of extracellular antioxidants’ potential in avoiding or delaying oxidative stress is being more debated. The original optimism for their good health impacts was based primarily upon in vitro studies. The first investigations ignored the antioxidants’ in vivo bioavailability, which was often fairly poor. The antioxidant’s significant in vitro organic compounds reactivity is thus not indicative of its efficiency in vivo. Furthermore, as demonstrated by independent reviews [67,68], antioxidant medication may be ineffective and even hazardous. Relevant results on alternative action mechanisms of antioxidant compounds are included in the article of Hrelia and Angeloni [69]. Their research demonstrates that natural antioxidants are extensively digested in vivo, causing their oxidation potential to decline significantly at the structural level. The researchers noted an increasing scholarly interest in the relations of natural antioxidants and proteins implicated in the intracellular signaling cascades, as well as gut macrobiotic regulation. Currently, in natural antioxidant research, (i) combination therapies that use the synergistic impact of naturally occurring antioxidants, (ii) anti-aging influence of fermented planning and preparation, (iii) enzymatic study, (iv) genome sequencing, (v) studies of the impact of antioxidant activity on the intestinal microbiota, and (vi) research on the determinants of antioxidants on the immune system are descriptions of research problems.

## 3. Mechanism of Free Radicals & Antioxidant

**Figure 1:**
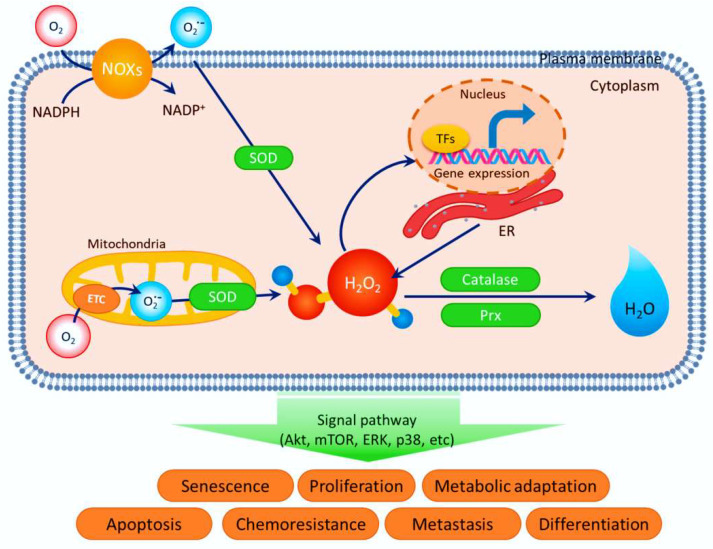
Redox homeostasis between generation and elimination of (ROS). Reproduced from the permission of ref. [81].

The fixed concentration of ROS and the rate at which such particles’ cells consists of three materials, according to studies, are completely related to the level of balance between conversations of ROS generation all across the body, the appearance of low-molecular-weight antioxidants, and the behavior of defending enzymes. There is a balance between both the development and regulation of ROS under normal conditions. A medical condition known as oxidative stress results from an imbalance in the number of antioxidants and free radicals in the body. The high level of ROS/RNS formation inside the body, as well as the movement in the imbalance of pro-oxidant and antioxidant components toward the “oxidation” process, leads to the enhancement of the rate of free radical responses. Oxidation causes damage to the brain as well as metabolic performance degradation; it commonly leads to apoptosis and, in severe cases, tumor formation. The oxidative stress of the molecular pathways contains DNA damage, the formation of genetic mutations, oxidation of fatty acids, proteins in living cells, alterations in protein function, and the promotion of apoptosis. It is also proves that oxidative further affects aging for the human. It needs to be noted which modest levels of ROS in human bodies are not dangerous. A high amount may induce a range of diseases, inflammation, and homeostasis modification [70,71,72]. The physiological contradiction of ROS/RNS action in the cell, as well as its role in cell respiration, is like a double-edged sword. ROS/RNS serve an important regulatory function; in addition, they are extremely toxic agents whose activity is at the root of many common diseases, modifications, and tumors. Therefore, the dosage of RNS or ROS in cells, which controls its mechanism of impact, explains this split of molecular processes. Many conditions are thought to contribute to oxidative in humans, the most prominent of which is abnormal behavior. This situation is typically caused by excessive stimulation of cells, tissues, or microorganisms for extra sources of RNS/ROS or by an increase in the rate of generation of an endogenous pool in ROS/RNS. The primary producers of reactive oxygen molecules and radical species are the biological metabolic systems essential for our bodies to function properly and to stay healthy. However, excessive exposure to damaging external chemical or physical stimuli can cause a considerable rise in ROS generation in cells [73,74,75]. The redox forms of N_2_ that include NO (II), as well the molecules designed from them, are a side effect of Ni-troxylanion (NO), peroxynitrite (ONOO), and metabolism. NO^+^ is an essential group of molecules with delocalized electrons associated with the high chemical activity. These chemicals protect the body against microorganisms under ecological factors. If there are problems in the creation of RNS, their levels rise excessively, resulting in nitrosation strain, a process related to oxidative strain. Nitrosylation for proteins occurs as a result of nitrosation stress, altering their structure and inhibiting their catalytic performance. Proteins with metal ions and their structure are extremely vulnerable to RNS activity. The association of RNS to such polypeptides alters hemoglobin, myoglobin, aconitase, and cytochrome c. Furthermore, RNS cause pathological abnormalities upon architecture for the cytoskeleton and transcription factors; their reaction with lipids causes peroxidation and changes in cell membrane permeability [76,77]. Antioxidants could attack free radicals by using one of three mechanisms: hydrogen atom transfer [HAT], single-electron transfer followed by proton transfer [SET-PT], and sequential proton loss electron transfer [SPLET]. Separating between these processes is difficult, because multiple mechanisms may be at work in different processes [78,79,80]. Other methods include spin entrapment and antiradical using antioxidant properties [78,80]. An antioxidant’s method of action is estimated by its structure, solubility, and media conditions, such as pH and solvent and temperature employed as represented in (Figure 1) [81].

## 4. Metal Chelates with Antioxidant Properties

The synthetic features for metal structures may plan to employ various metals and ligands, consequently enabling its progress for detailed uses. The production and estimation of the performance of chelates as physiologically active ligands has increased in popularity over time [82,83,84]. Combining redox characteristics for metal ions and various ligands is a viable technique for developing antioxidant molecules with several modes of action [85,86]. In general, antioxidants that promote the defense against free radicals in the body are typically obtained via dietary consumption [87]. Organic substances with strong antioxidant capacity, such as flavones, flavonoids, phenolic acids, and cinnamic acids, are abundant in seeds, fruits, vegetables, wine, and tea, among other natural products [88,89]. Such antioxidants have a COOH or OH, and an oxo group for flavonoids and flavones, in their structural system, as well the ability to coordinate towards various metal ions and developing stable chelates, because most of these materials are associated with the metal as chelates via oxygen atom complexion [85,90]. Complex formation through natural products is favored towards elements with lower oxidation and spins levels, because the occurrence of phenyl ring through (1) or (2) chains in its molecules allows bond-strengthening back donation. Molecular coordination for the antioxidant ligand to a central metal ion enables the control of all features through the following benefits: (1) complexation for the substituent of control solubility, and (2) stability through the phenoxyl intermediate, produced in the substituent electrochemical process with the assistance of the metal ion [86]. Flavonoids, hydroxycinnamic acids, polyphenols, flavones, and carotenoids contain heterocyclic structures with UV-vis absorption at 380 nm. The combination of organic ligands causes the spectrum to move to the red region, indicating the forming of a complex. Additionally, such antioxidants are luminous, and the difference in emissive qualities between both the free ligand and the synthesized chelate may be used to determine coordination [91]. Organic Schiff bases are commonly utilized in the production of coordination with antioxidant effect, in addition to coordinating bioactive ingredients [92]. The reaction of condensation between aldehydes and primary amines produces the RN = CH − R′ group, while both R and R′ are substituent function groups connected to the cores. Because the azomethine group’s N_2_ atom exhibits sp^2^ hybridization, it has a pair of electrons available. Furthermore, the presence of the double bond, which has an electronic donating feature and nitrogen’s low electronegativity, makes this azomethine group atom a site with high electronic density donation, therefore the Schiff bases are good ligands for the chelation with metal centers [93,94]. Complex formation through Schiff bases and natural antioxidants has the sequence advantages: (1) rapid and simple synthesis; (2) various methods of mechanisms; (3) the ability to use chelates as living mechanisms for antioxidant performance, and (4) significant growth in antioxidant activity relative to the complex’s free ligand [84,91]. Because of their antioxidant effects, metal lophthalocyanines have received increased attention. Due to the attendance of four aromatic sp^2^ N_2_ atoms, phthalocyanines are aromatic and macrocyclic ligands are accepted for efficiently matching for various metal centers as a chelate while the complex is formed. Furthermore, these ligands feature a conjugated 18 *p*-electron process, which gives them chemical resistance and stability as antioxidants [95]. Phthalocyanines have a planar structure and are poorly soluble. To operate as antioxidants, phthalocyanines must be successfully fabricated to enhance their solubility, and these alterations did not affect their crystallinity. A great number of sequences of metal lophthalocyanines were reported in this regard. The position of the metal center of reference to the ligand’s mean plane governs the structural arrangement for this family of metal complexes [96,97]. The majority of the items in this article are chelates containing natural products, phthalocyanines, and Schiff bases. Several samples of nanoparticles are also presented to display why inorganic materials in general may be used as antioxidant compounds in a variety of ways. Moreover, samples of chelation improving, or not improving, antioxidant activity are shown throughout. Furthermore, studies of chelation improvements, or degrading antioxidant performance, are provided throughout this article. As can be seen, the variation in antioxidant activity of free to coordinating ligands is driven by the presence of metal, oxidation state, shape, and how the ligands link to the central metal atom.

## 5. Biological Outcomes of Oxidation by ROS

It is expected that all cells are treated for potentially detrimental effects every day as a result of ROS activity. The action of antioxidants inhibits the source of endogenous or exogenous reactive oxygen species under balance conditions. The organism performs effectively as long as these are in balance. However, where there is a growth in oxygen free radicals or a drop in antioxidant potential for some reason, a situation identified as oxidative stress arises. Many problems can develop as a consequence of such a diseased status. A lot of radicals can affect both (tissues and organs). Conversely, free radicals affect proteins, which are critical for our cells’ effective functioning, including fatty acids, peptides, and sugars. Additionally, ROS causes Mutations in DNA and genetic instability [98,99,100].

### 5.1. Reactive Oxygen Species and Lipids

**Figure 2:**
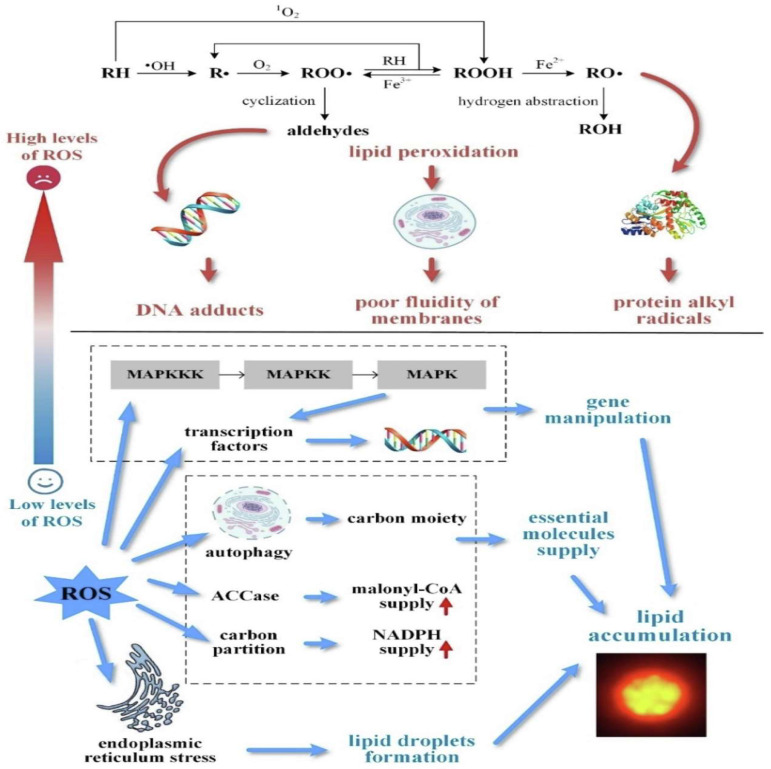
Lipid peroxidation and accumulation mediated by ROS. R: hydrocarbon chain & R·: lipid alkyl radical & RH: the fatty acid in lipid & ROOH: hydroperoxy fatty acids & ROH: lipid hydroxide & ROO·: lipid peroxyl radical & RO: lipid alkoxyl radical & MAPK: mitogen-activated protein kinase & MAPKK: MAPK kinase & ACCase: Acetyl-CoA carboxylase & MAPKKK: MAPKK kinase. Reproduced from the permission of ref. [108].

The most typical oxygen radicals’ reaction interacting with cell function is cyclic membrane degradation, which involves the degradation of polyunsaturated fat radicals found in membrane lipids and lipids. The hydroxyl radical is the primary radical that damages the lipids of cell membranes during non-enzymatic peroxidation of lipids. The H_2_ atom is disengaged from the unsaturated lipid structure of liposomes in the first stage of initiation, and alkyl radicals (L^•^) are generated. Double bonds are rearranged, and conjugated bonds are formed as a result of the process. Signal amplification alkyl radicals can then combine with O_2_ or fatty acids, resulting in the generation of further aliphatic alkali radicals or fat peroxyl radicals (LOO^•^). It is a cyclic process that may result in the auto-oxidation of hundreds of polyunsaturated fat molecules. The following step is to inhibit peroxidation that can occur in a de-oxygenation reaction involving two lipid alkyl/peroxyl radicals or two separate radicals [101,102]. Fatty acid dimers and oxo/hydroxy fatty acids with altered and degraded structures are formed during the lipid peroxidation reaction. Further hydrolysis-associated gadgets produce highly reactive aldehydes and hydroxyl aldehydes, such as MDA [malondialdehyde] and 4-HNE [4-hydroxy-2-nonenal]. Secondary peroxidation transmissions are particles that interact with peptide amino acid residues, thereby modifying their structure and function. It is especially significant in signaling networks, where conformational changes in amino acids depending on MDA or 4-HNE frequently result in preventing or stimulating the performance of several crucial enzymes in the route. Furthermore, 4-HNE and MDA interact with the nitrogenous bases of DNA, producing chain breakage and preventing DNA replication [103,104,105]. These extremely chemical oxidative intermediates have been demonstrated to decrease antioxidant capacity and impair antioxidant enzyme activity. Furthermore, unsaturated aldehydes are involved in many channels of cell signaling or metabolic process regulation, contributing to their disruption by blocking or promoting the activity of multiple enzymes. Mitochondria are especially susceptible to oxidative damage. Changes in the oxidation of the mitochondrial matrix impair the electron transference sequence and enhance ROS generation [106,107]. Figure 2 represents Lipid peroxidation and accumulation mediated by ROS [108].

### 5.2. Reactive Oxygen Species and Proteins

Structured and enzymatic peptides, in addition to liposomes, are sensitive to reactive oxygen species. Peptide oxidation is mostly caused by the most reactive hydroxyl radical, though other peptide modifications, including thiol bond oxidation, can happen in the presence of singlet oxygen with peroxides. Oxidative stress involving ROS is related to fat peroxidation in that it involves the polynucleotide strand and amino residues, though it is not always a chain process. The lipid oxidation behavior of free radicals causes several undesirable structural changes in proteins, such as the hydroxylation of aliphatic and aromatic amino acid sequences, the structure of nutrient hydroperoxides, the oxidation of thiols and methionine residues, the transformation of certain cysteine residues in and out of c-o derivatives, the transformation of the peptide bonds, and the forming of the bridge inside for the same protein molecules. The hydroxyl radical contributes to the initiation of the peptide chain’s oxidation process. It induces the hydrogen ion on the carbon of the amino acid to separate. The produced aliphatic radical interacts vigorously using oxygen to produce aliphatic hydroperoxide. That molecule may be transformed into such an alkyl radical, which would be directly responsible for the splitting of protein molecules. The alkyl, alkyl peroxide, and alkyl radicals may react with some other residues of amino acids within the same or other associated proteins, generating more charges. It is now widely accepted that chemical modifications in proteins mediated by ROS oxidation can occur at each amino acid position. The heterocyclic proteins tyrosine and tryptophan, as well as cysteine and methionine, are among the most sensitive to free radical destruction. Peptide modification induced by ROS has been shown to develop in the genesis of a variety of diseases, as well as throughout the process of aging. Vital amino acids in enzymes and signaling molecules are regularly destroyed. The loss of a protein’s physiological properties seems to be the most common result of structural changes that can lead to the restriction of important enzymatic activity or irregularities for proteins having regulation throughout transcriptional [109,110,111]. An increase in ROS-dependent protein oxidation has been shown to correlate with microorganism age. However, it is vital to highlight that a rise in the proportion of cell damage proteins is a component of several age-related pathologies. While transformed peptides are less sensitive to proteolysis, they accumulate in cells, causing necrosis in some cases [112,113]. Various investigations have also found that oxidative damage to polypeptides and the proportion of their reduced derivatives have a substantial role in the progression of cardiac disease [114], particularly atheroma and diabetes, as well as neurological disorders such as Alzheimer’s and Parkinson’s. Aside from degraded fatty acid LDL fractions, oxidizing altered proteins were identified in the aqueous deposits of diabetics with atherosclerosis. Free radicals have an important part in the development of neurodegeneration. Neurons are particularly vulnerable to oxidative stress due to their accelerated oxygen consumption and high content of unsaturated fats. Proteins are threatened by the combination of extremely high metabolic and aerobic rates. The coupling of very high metabolic and respiratory activity in brain cells affects proteins. Many factors, including abnormal proteins and their accumulation, as well as abnormalities in transcription factors caused by ROS, have been implicated in the pathogenesis of dementia [115,116,117].

### 5.3. ROS and DNA

**Figure 3:**
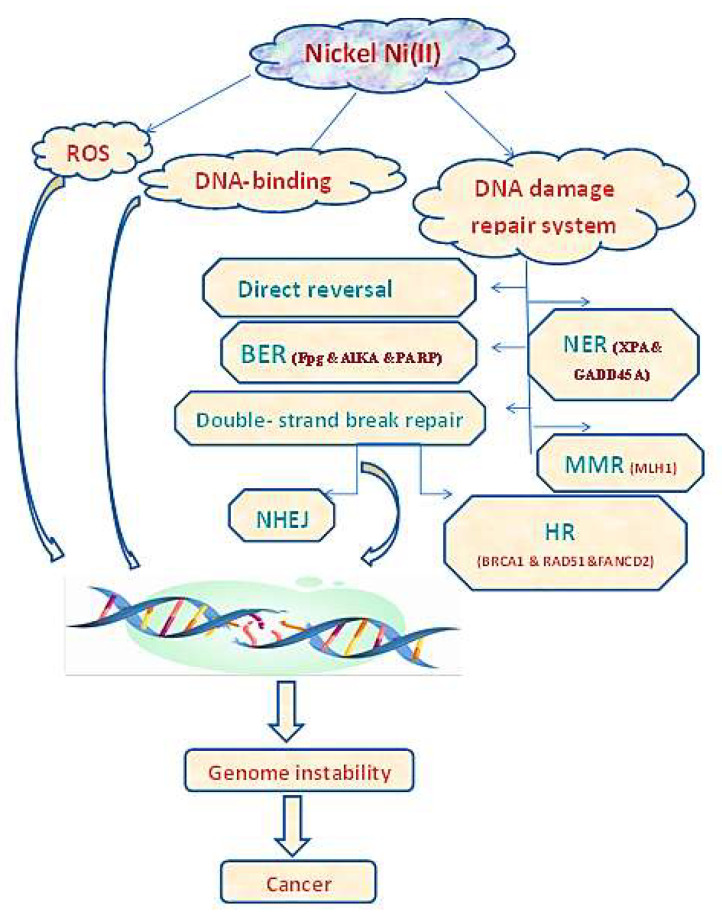
A simplified model of Ni-induced DNA damage in the onset of cancer. Excessive exposure to Ni can induce DNA damage, mainly through direct DNA binding and ROS generation. Ni can also repress the DNA damage-repair pathways, including direct reversal, BER, NER, MMR, HR, and NHEJ repair. DNA damage causes genome instability that may ultimately contribute to cancer. Reproduced from the permission of ref. [124].

Nucleic acids have increased constancy as well as tolerance for free radicals. After destruction, such cell membranes recover efficiently, while mutated nucleotide sequences are extracted and removed from the Target DNA. The OH radical, which can induce free radical destruction through practically all nucleotide segments, is the most dangerous hazard to our DNA. Nitrogen bases, sugar residues, and phosphodiester bonds can all be oxidized, resulting in nucleotide alteration, DNA damage, and even strand breaking. Thymidine is highly vulnerable to OH radical action, which destroys it and converts it into thymidine dimers and peroxides. The interaction of guanine nucleobase through the OH radical, which results in the creation of 8-hydroxyguanine, is likewise extremely hazardous. It is important to note that the prominent impact of ROS on DNA, as well as the adjustment of nitrogen bases and deletions, or genetic changes, were observed in disorders of transcription factor association, growth factor of primitive, genetic code cuts, and a variety of other lethal cell oddities [118,119,120]. Genetic Variation is more sensitive to RFT because it lacks intron sequences and is not covered by histone proteins. Genetic Code displayed a substantially higher sensitivity to free radical destruction than to nuclear DNA, straight through endogenous ROS formation. This is critical for the development of progressive disorders, where oxidative stress-induced metabolism is observed in the majority of instances [121,122,123]. Figure 3 represents a scheme of metal-induced DNA destruction in cancer existence. Extreme contact with metals such as Ni may induce DNA to destroy, mostly through DNA binding and ROS generation. Ni could also suppress the DNA destruction repair paths, with direct reverse, BER, MMR, NER, NHEJ, and HR repair. DNA destructions cause gene instability that may eventually contribute to cancer existence [124].

## 6. Main In Vitro Assays to Assess Antioxidant Activity

### 6.1. DPPH Radical Assay

The DPPH radical is stable and has a delocalized electron, which confirms a purple color with an absorption maximum at 517 nm, and is detectable through EPR [125,126]. A limitation of this method is the steric hindrance between DPPH and the antioxidant molecules [127]. Since the radical site is located in the center of the molecule, smaller antioxidant molecules have easier access to this point, resulting in greater activities compared with those of larger molecules. Due to this fact, to verify the reaction between the antioxidant and DPPH, the reaction solution must be kept in the dark for 45 to 60 min to ensure that the process occurs [128].

#### UV–Vis Detection

The colorimetric method is based on the ability of a given antioxidant to reduce the DPPH radical to hydrazine, changing the color of the solution from purple to yellow [128]. Scavenging of the DPPH radical by an antioxidant is detected by UV–vis spectroscopy by monitoring the DPPH absorption band at 517 nm [125,127]. The reaction medium for this assay should preferably be alcoholic (ethanol or methanol) to avoid processes of aggregation of the stable radical. The use of water should be minimized because it can favor aggregation, with no aggregation problems observed up to the 1:1 (ethanol:water) ratio [129]. The main limitation of the method lies in the absorption range of the DPPH radical, which occurs in a visible region where several antioxidants also absorb, possibly hindering the detection of the end of the reaction between DPPH and the antioxidant [130]. Nevertheless, DPPH is a simple, accurate, reproducible method. DPPH is commonly used to evaluate the effectiveness of metal complexes containing flavonoids with antioxidant properties.

### 6.2. Lipid Peroxidation

Lipid peroxidation is the process under which free radicals attack the side chain of unsaturated fatty acids. The radical abstracts a hydrogen atom from close to the double bond of the fatty acid, forming a conjugated diene. The conjugated diene is an unstable lipid radical that can react with oxygen to form the lipid peroxyl radical (ROO·) [131]. Because this is a process of oxidation of fats, saturated fatty acids are more resistant to radicals than polyunsaturated fatty acids.

#### Lipid Peroxidation—EPR Detection

Lipid peroxidation can be performed using electron paramagnetic resonance (EPR) assay by monitoring the signal of the adduct formed between the radical species and the spin scavengers, as for the Lipid Peroxidation Inhibition Capacity, Superoxide Radical Anion (O^2−^), Hydroxyl Radical (OH^−^), and Singlet Oxygen (^1^O_2_) assays. EPR can also be performed to follow the DPPH^•^ and ABTS^•+^ signal suppression in the radical scavenging method. The advantage of EPR over spectrophotometry is that, in the former, the color of the antioxidant does not interfere with the measurement or detection of the results, whereas in the latter, the color may hinder detection in the UV–vis region and lead to misunderstandings in the quantification of antioxidant activity.

Recently, Marchi et al. used this technique to measure the in vitro extension of lipid peroxidation of pork muscles in the presence of the [Mg(phen)_2_(iso)]^+^ (Mg-iso) complex, where phen = 1,10-phenanthroline and iso= iso vanillic acid. The lipid radicals form stable spin adducts with 4-POBN, giving rise to the detected signal that corresponds to three doublets. The hyperfine coupling parameters of the formed adducts associated with the ROO^−^ radical are aN = 15.77 G and aH = 2.86 G. Under severe conditions (presence of atmospheric O_2_ at 75 °C), the Mg-iso complex was able to scavenge the lipid radicals from both Longissimus lumborum and Psoas major muscles of fresh minced pork [132].

### 6.3. Ferric Reducing Antioxidant Power (FRAP)

This method is based on the ability of an antioxidant to reduce a Fe(III) complex to a Fe(II) complex containing the 2,4,6-tri (2-pyri dyl)-1,5,5-triazine ligand (TPTZ) through the SET mechanism [133]. The reduction of [Fe(TPTZ)_2_]^3+^ to [Fe(TPTZ)_2_]^2+^ is accompanied by a color change from light to dark blue. Formation of the reduced complex is detected by UV–vis spectroscopy by monitoring the absorption band at 593 nm [134]. In this methodology, the reaction medium is acid (pH = 3.6) to maintain the solubility of the Fe(III) complex. A reaction medium with pH > 7.0 may favor the formation of Fe(OH)_3_, which is an insoluble solid. FRAP is a simple, rapid, low-cost method [132].

### 6.4. Cupric Reducing Antioxidant Capacity (CUPRAC)

The CUPRAC method is based on the ability of an antioxidant to reduce a Cu(II) complex to a Cu(I) complex containing 2,9-dimethyl-phenanthroline (men) as a ligand through the SET process. This is a stable and rapid method. The reduction in [Cu(dmphen)_2_]^2+^ to [Cu(dmphen)_2_]^+^ is accompanied by a change in color from blue to yellow for the reduced complex. The formation of the reduced complex is detected by UV–vis spectroscopy by monitoring the absorption band at a wavelength of 450 nm [135]. The Cu^2+^ ion has a redox potential (0.16 V) lower than that of the Fe^3+^ ion, which makes the CUPRAC reaction more selective than the FRAP reaction [136]. Unlike FRAP, this method is performed in an aqueous solution with neutral pH (7.0–7.4) or ethanol [136]. Thus, this assay can be applied to a greater variety of antioxidants with hydro- or lipo-soluble chemical structures [132].

### 6.5. H_2_O_2_ Scavenging Activity

For the free radical scavenging activity using hydrogen peroxide, a 2 mg/mL sample of DMSO was taken and added to 40 mM of hydrogen peroxide in phosphate buffer (pH 7.4). A phosphate solution without hydrogen peroxide was used as the blank and the absorbance was measured at 230 nm. The percentage of radical scavenging effects was calculated general equation in the DPPH method [137].

### 6.6. ABTS Assay

The ABTS assay was performed as previously described [138]. The reagent was prepared by mixing aqueous solutions of ABTS (c = 7 mmol·dm^−3^) and K_2_S_2_O_8_ (c = 2.45 mmol·dm^−3^) in a volumetric ratio of 1:1; and left for 12–16 h at T = 296.15 K to generate ABTS^•+^ cation radicals. Then, 1 cm^3^ of the obtained ABTS^•+^ solution was mixed with 60 cm^3^ of methanol. An amount of 1 cm^3^ of ABTS^•+^ solution and 1 cm^3^ of methanolic solution of tested compounds (prepared at different concentrations, as described before) was mixed and incubated for 7 min at T = 296.15 K. The control contained methanol instead of the sample. The absorbance of the solutions was measured using the UV-VIS spectrophotometer at λ = 734 nm against methanol (the reference). The ability to scavenge ABTS^•+^ cation radicals was expressed as the percentage Inh ABTS^•+^ [139].

## 7. Overview of Metal Chelates as an Antioxidant

**Figure 4:**
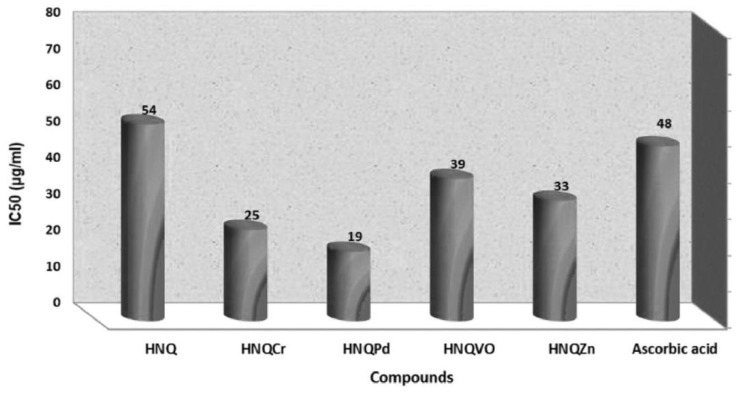
The antioxidant performance of HNQ (1) imine ligand and its Cr(III),VO(II), Zn(II) and Pd(II) complexes (2–5). Reproduced from the permission of ref. [140].

**Scheme 1:**
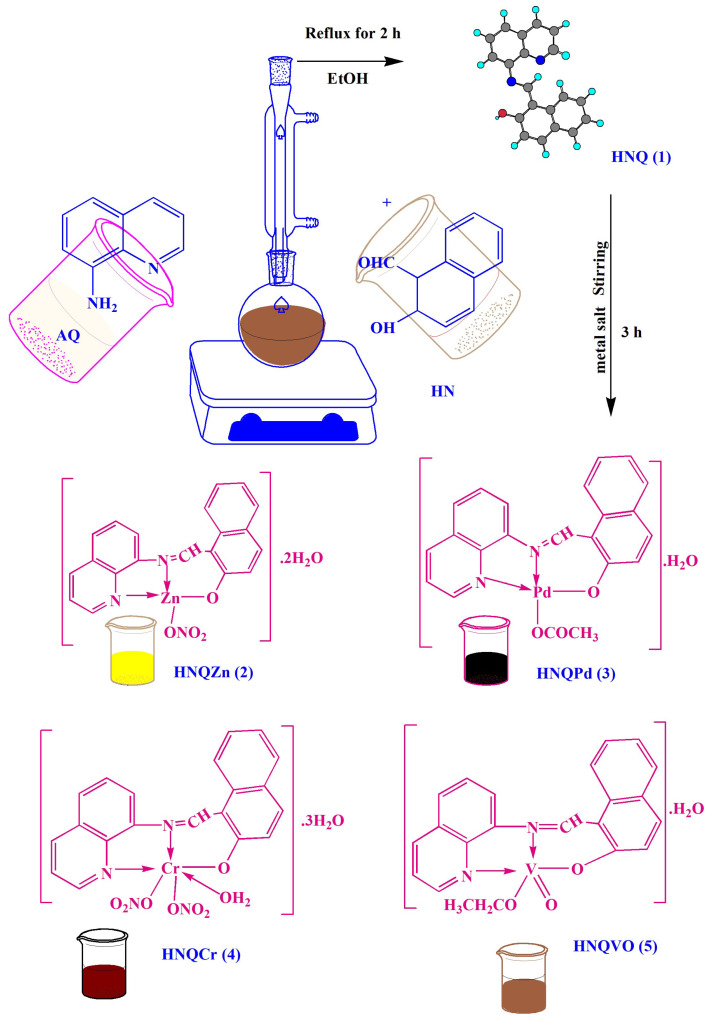
Diagram of synthesis pathway for new HNQ (1) imine ligand and its metal chelates (2–5). Reproduced from the permission of ref. [140].

Aljohani et al. (2022) prepared a new sequence of bioactive series, through the novel Schiff base ligand HNQ (1-Quinolin-8-yliminomethyl-naphthalen-2-ol) (1). CHN, conductivity, IR, magnetic moment, NMR, TGA, as well UV-Vis, were applied to explain the chemical structure. Due to this, a proper geometry had been planned for each chelate as shown in (Scheme 1). In addition, the antioxidant performance of the tested compounds (1–5) was studied in vitro, and the data exposed that the ligand’s performance was comparable to which of the standard drug, as shown in Figure 4. This assay was reinforced through various theoretical studies. Biological reproduction was implemented by Pharmit link to examine the drug-like molecules and to determine the degree of binding with DNA-protein (1 bna). Moreover, MOE-docking was applied to put a perfect view of the contact features among the tested compounds and 1 bna protein. The model study exposes the importance of the HNQ (1) ligand towards the DNA and the insignificant role of chelates, which was estimated. When associating in vitro through in silico data, this variance was observed, which may propose that an occurrence of any indefinite process enhances the performance of chelates in living cells [140].

**Scheme 2:**
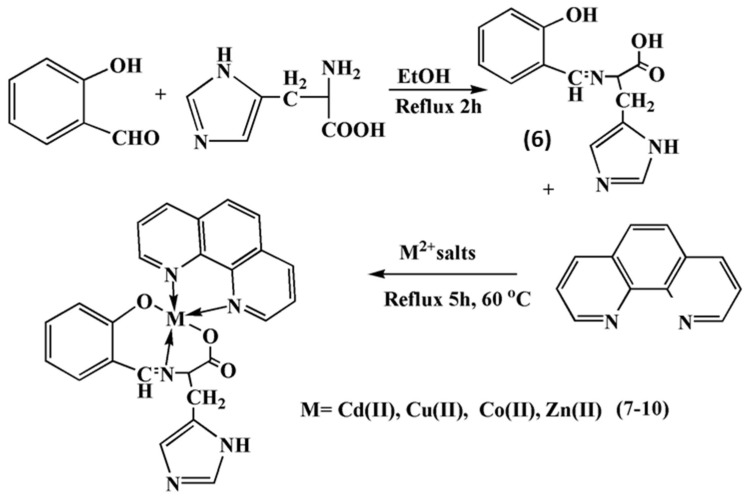
Synthetic protocol of Schiff base ligand (6) and their metal complexes (7–10) [137].

Sumathi S (2022) prepared an organic ligand through the interaction of amino acid (L-Histidine) with salicylaldehyde and 1, 10 phenanthroline (6), as well its metal chelates of [ML1L2] 7–10 (wherever M = Cd(II), Cu(II), Co(II), and Zn(II)) prepared as presented in (Scheme 2) and were screened for in vitro antioxidant activity through DPPH and H_2_O_2_ tests. The obtained data are displayed in Table 1. Cd^2+^ chelate had the maximum antioxidant scavenging performance among the tested metal chelates with 86.06% and 84.64% through the DPPH and H_2_O_2_ methods, respectively [137].

Nongpiur et al. (2022) studied the reaction of [(arene)MCl_2_]_2_ through bidentate 4-, 5-diazafluorene-9-one (dafo) and resulting organic ligands (L1–L3) (11–13) in the existence of (NH_4_)[PF_6_] yielded cations chelates having general formula [MLCl(arene)]PF_6_ {M = Ru, arene = benzene (14, 16, 18); M = Ru, arene = *p*-cymene (15, 17); M = Rh, arene = Cp* (19, 20, 21); M = Ir, arene = Cp* (22, 23, 24); [4,5-diazafluorene-9-one (L1) (11), N-(4,5-diazafluoren-9-ylidene)aniline (L2) (12), N-(4,5-diazafluoren-9-ylidene)phenyl hydrazine (L3) (13)] (Scheme S1).To evaluate the biological efficacy of tested compounds, antioxidant experiments were screened. The tested compounds also had significant antioxidant action against DPPH radicals, according to the results. Antioxidants could interact with extra free radicals by meddling through the oxidation procedure, similarly through substitutes as sensitive type scavengers. DPPH scavenging technique is extensively used to control antioxidant performance. The radical scavenging capability (%) for the tested free ligands and their chelates was identified as related to the radical scavenging outcome by DPPH with ascorbic acid as a reference; the values are shown in Table 2. The data displays that the tested compounds showed noticeable radical scavenging performance [141].

Priya J and Madheswari D (2022) developed a new organic ligand (25), in combination with the usage of less-expensive elements, through significant therapeutic potential and have prompted huge interest in the growth of organic ligands. Thus, four various metal chelates comprising Mn^2+^ and Ni^2+^ and Cd^2+^ and Pb^2+^ (considered as chelates 26–29) were prepared through a new tetra-dentate organic ligand (L) (25), obtained through condensation of (3,5-dichlorosalicylaldehyde and trans-1,2-diaminocyclohexan, as proven here (Scheme S2). Research on the free radical scavenging applied by the ligand L (25) and the studied metal chelates (26–29) showed that the Ni(II) chelate had more effectiveness. The antioxidant values are displayed in Table 3 [142].

Elaaraj et al. (2022) prepared novel metal chelates of Zn^2^, Co^2+^, Ni^2+^, and Cu^2+^ derived from the ligand 2-(thiophene-2- yl)-1-(thiophen-2-ylmethyl)-1H-benzo [d] imidazole. The antioxidant performance for tested ligand, as well the tested chelates estimated by DPPH process, were compared to the standard antioxidant. The data obtained exhibited that the antioxidant performance of the tested ligand, as well as their chelates, were moderate and that the Cu(II) chelate had a great performance, outdoing ascorbic acid. We could determine that the chelation of the tested ligand stimulated the antioxidant performance. The antioxidant performance considerably improved for the electron-withdrawing effect of the M^2+^ ion, which simplified the issue of H_2_ to decrease the DPPH radical [143].

**Figure 5:**
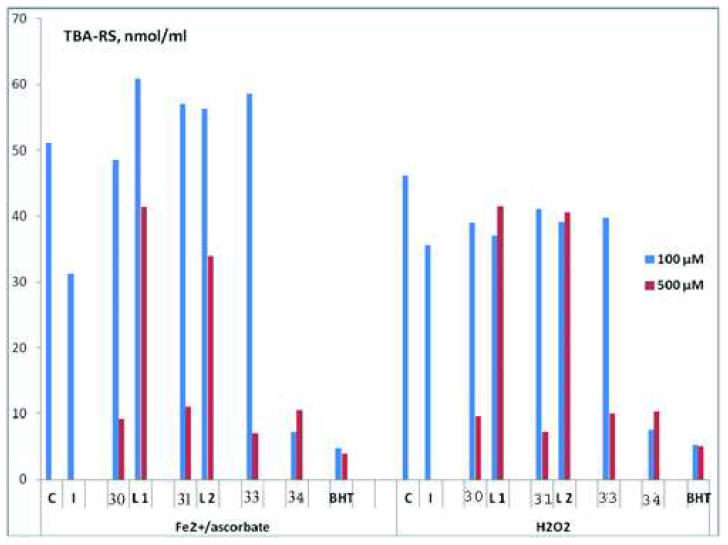
Antioxidant performance of the test compounds (Cu chelates 30–33, ligands L_1_ (34) & L_2_ (35)) at concentrations of 100 and 500 μM. C—Control without test compounds. I—intact samples (without initiated oxidation). BHT—standard antioxidant 2, 6-di-tert-butyl-4-methylphenol. Reproduced from the permission of ref. [144].

**Figure 6:**
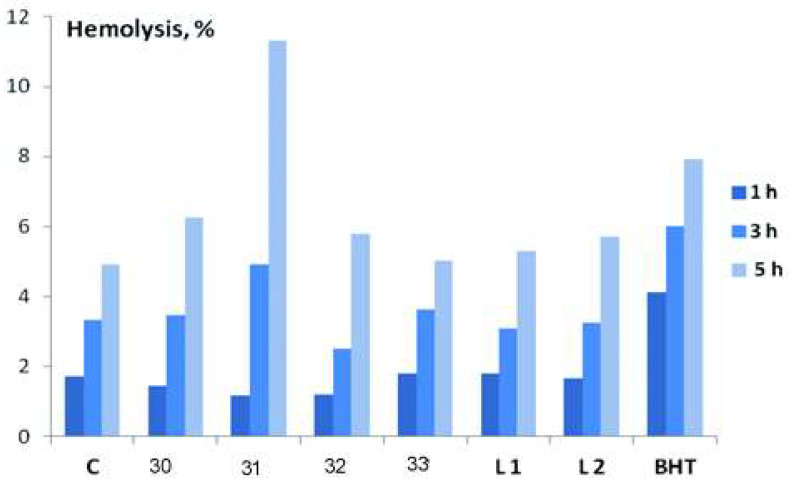
Hemolytic activity of the test compounds (Cu chelates 30–33, ligands L1 (34) & L2 (35)) at a concentration of 10 μM after 1, 3, and 5 h of incubation. C—control without test compounds. BHT—standard antioxidant 2,6-di-tert-butyl-4-methylphenol. Reproduced from the permission of ref. [144].

Gur’eva et al. (2022) prepared a new substance from Cu(II) chelates (30–33) with terpene products of ethylenediamine (34–35) (Figure S1), the results of reviewing their antimicrobial and antioxidant performance in vitro are debated. All calculated Cu(II) chelates (30–33) exhibited considerably greater antifungal action than the strains of S. salmonicolor, C. Albicans, P. notatum, which were associated with the motion of the scientific antifungal amphotericin drug. Great antibacterial performance for Cu(II) chelates with terpenes of ethylenediamine was demonstrated in relation to the S. aureus strain (MRSA), which is strong against the standard antimicrobial ciprofloxacin. Via several experiment systems, a relative calculation of the antioxidant performance of the prepared Cu(II) chelates and the organic ligands was accepted. As shown in Figure 5 the salen-type chelate four had the maximum antioxidant performance in the typically introduced oxidation for a substrate covering lipids greater for other Cu(II) chelates in terms of the capability of keeping erythrocytes below surroundings of H_2_O_2_-induced hemolysis. As shown in Figure 6 [144].

Devi et al. (2022) prepared multiple Cu(II), Co(II), Zn(II), and Ni(II) chelates through four Schiff organic ligands (BHAP) (36), BHACM (37), BHACN (48), and BHIMP (39) gained through the reaction of 4-(benzyloxy)-2-hydroxybenzaldehyde with several aminophenols and were considered through many spectral techniques as represented in Scheme S3. The tested substances (from 36 to 55) were estimated for their antioxidant performance in vitro then found that the prepared M^2+^ chelates were very powerful, displaying proficiency for decolorizing the purple solution of DPPH related to free ligands. Cu-chelates had the highest potency, with IC_50_ data as 2.98 toward 3.89 μM range. As represented in Table 4, the MOD of organic ligand BHACM (37) and its copper-chelate with enzyme; C. Albicans sterol 14-alpha demethylase recommended the hydrophobic binding. Moreover, in silico test strained which meant the tested materials could be utilized as orally active drugs [145].

Ali et al. (2022) synthesized heterocyclic chelates of Cr(III) and Fe(III) by reducing succinic dihydrazide with 5-chloroindoline-2, 3-dione in an aqueous medium through the original technique in (1:1:1) proportion with M.W irradiation system (Scheme S4). The tested chelates were strained toward bioactivity. Anticancer performance was assessed in relation to the HNSC cells line and antioxidant performance is completed through the DPPH test. All tested compounds (56–59) exhibited free radical scavenging activity. [Cr(C_12_H_10_N_5_O_2_Cl) (NO_3_)_2_]NO_3_ chelate presented maximum free radical scavenging activity (IC50 ≥ 50 μg) between all of the studied chelates in association with BHA (IC_50_ ≤ 50 μg). An in silico test was completed through MOD with EGFR tyrosine kinase. The results displayed which tested materials had substantial anticancer and antioxidant performance [146].

**Scheme 3:**
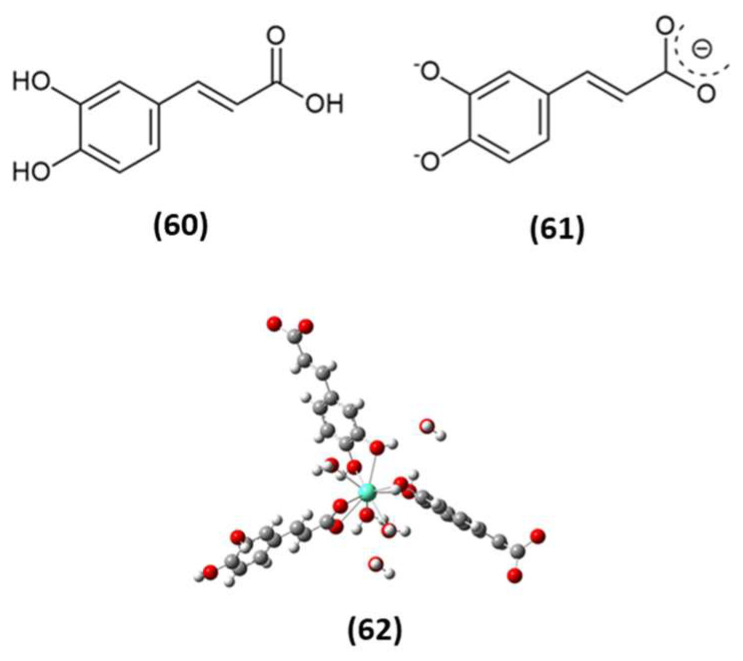
Chemical structure of CFA in its fully protonated (CFA, LH_3_) and deprotonated (L^3−^) forms: caffeic acid (CFA, LH_3_) (60); caffeinate (L^3−^) (61) Eu(III)-caffeinate metal complex (62). Reproduced from the permission of ref. [139].

**Figure 7:**
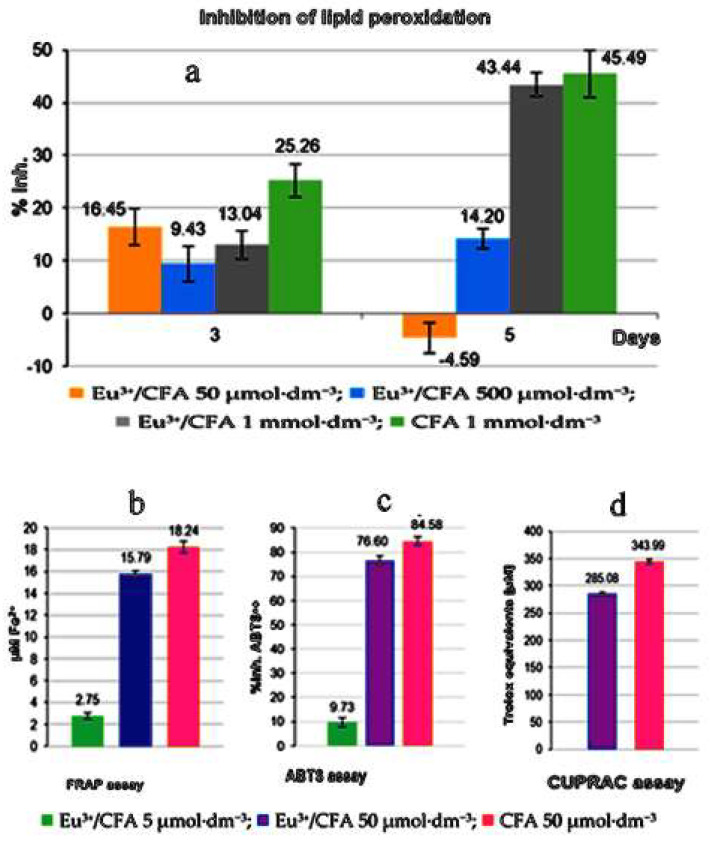
(**a**) % of reduction in oxidative stress by Eu^3+^/CFA complicated at 50, 500, and 1000 mol dm^−3^; (**a**) CFA and Eu-CFA antioxidant properties assays at the identified concentration level (**b**) FRAP assay assessment of reducing possibility; (**c**) proportion of inhibition of ABTS^•+^ cation radicals; (**d**) CUPRAC test findings demonstrated as Trolox offerings [mol·dm^3^]. Mean results from three independent tests ± SD are shown. Reproduced from the permission of ref. [139].

Arciszewska et al. (2022) said that caffeic acid (CFA(60)) and its anion caffeinate (L3−) (61) are just one of many bioactive components and chemo-preventive agents based on human nutrition. Their metallic chelates are also vital in living processes. However, research on the characteristics of CFA with inorganic metals is extremely uncommon, thus very little live or ecological data is revealed about all of these operating processes. The preponderance of their property, including physiological development and environmental influence, depend greatly on their structure, stability, and solution behavior. These interactions for the Eu(III)/CFA chelate were investigated using a multi-analytical-system strategy. The main molecular formula of the investigated metal chelate in the solid state was [Eu(CFA)_3_ (H_2_O)_3_]·2H_2_O (M:L ratio 1:3) (62), though the 1:1 forms were discovered in an aqueous medium at the optimal pH of 6 pH 10 ([Eu(CFA)] and [Eu(CFA)(OH)]) (Scheme 3). Electrochemical mobility tests were performed to analyze the interaction of Eu(III)/CFA (62) and CFA primarily across the cellular membrane. Multiple antioxidant studies have indicated that Eu(III)/CFA has less antioxidant action than pure CFA ligands. As a result, we used five free antioxidant techniques to demonstrate that Eu(III) did neither disrupt nor diminish CFA’s antioxidant effects (Figure 7). The IC_50_ for Eu(III)-caffeinate could not be determined using the DPPH approach because it precipitated at a higher concentration. In CUPRAC and FRAP tests, Eu(III)/CFA chelate showed decreasing assets, although lower than CFA (CUPRAC assay: 285.08 and 343.99 Trolox equivalence; FRAP biomarker: 15.79 and 18.24 mo·dm^3^ of Fe^2+^ for Eu(III)-caffeinate and the CFA chelate and CFA, respectively [139].

MedetalibeyogluH (2022) studied and synthesized the antioxidant activity of (EPM)2-ethoxy4-[(5-oxo-3-phenyl-1,5-dihydro-1,2,4-triazol-4-ylimino)-methyl]-phenyl-4-meth oxybenzoate (Scheme S5). The tested EPM (63) material was positively prepared through new resultant naturally significant 1-, 2-, and 4-triazole ligands. This study of 1-, 2-, and 4-triazole ligands had extended from the condensation of 3-phenyl-4-amino-4, 5-dihydro-1H-1, 2, 4-triazole-5-one and 2-ethoxy-4 -formylphenyl-4-methoxybenzoate. The antioxidant activities for the investigated organic ligands were estimated by applying the Dinis, Oyaizu, and Blois methods. The studied metal chelating performance for the novel prepared ligand and reference detected for the reduction in the order of α-tocopherol < EPM < EDTA was in agreement with the Dinis process. The tested synthesized organic ligand exhibited NLO stuff, which was 34 periods, as considerable as the feature of urea. The ability of H_2_ or e^−^ donation for EPM and Ascorbic acid, such as BHA, BHT, and α–tocopherol was projected through the DPPH. In this respect, the result exposed that the tested prepared EPM had not reported effective activity as a radical scavenger and did not have H_2_ donor performance. The EPM results demonstrated a definite Fe-binding potential, suggesting that their purpose as peroxidation protections might be linked to their iron-binding ability. The studied metal chelating performance of EPM and standards was studied toward decline into the demand of α-tocopherol < EPM < EDTA, which were 39.0%, 53.2%, and 85.7% at the final absorption, correspondingly [147].

Damena et al. (2022) studied novel [Co(L)(Cl) (H_2_O)_2_] (65) and [V(L)(O) (H_2_O) (SO_4_)] (66) chelates prepared from an (E)- 2-(((2-((2-hydroxyethyl)amino)quinolin-3-yl) methylene) amino)ethan-1-ol ligand C_14_H_17_N_3_O_2_ (64), CoCl_2_·6H_2_O and VOSO_4_ in Me OH solutions (Scheme S6). The antioxidant action of prepared ligands, as well as their metal chelates, were evaluated in vitro through DPPH. The organic ligand displayed fewer in vitro antioxidant performances than the tested chelates, whereas the Co chelate had a superior antioxidant performance through half-inhibitory concentrations (IC_50_ of 16.01 μg/ mL) than the free ligand, (VO) chelate. MOD study also recommended a lot of attention to the biological performance of the tested Co and VO chelates. Accordingly, molecules that have an antioxidant action could decrease the absorbance at 517 nm; this is related to the DPPH assay shifting hue during the recombination process [148].

**Figure 8:**
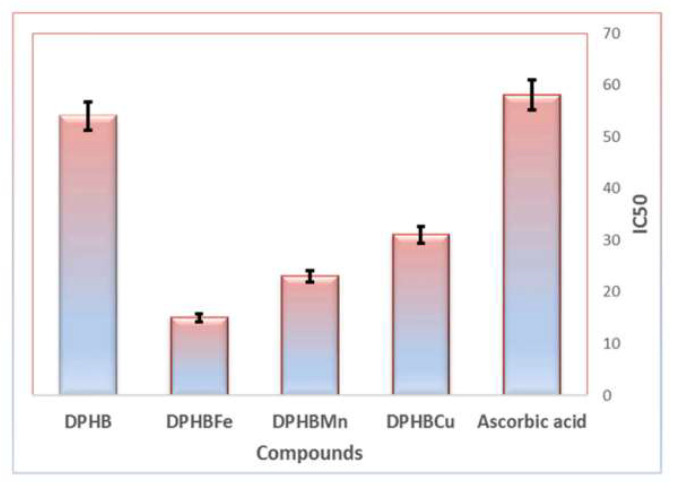
The inhibition performance of DPPH radical for DPHP (67) ligand and its complexes (68–70). Reproduced from the permission of ref. [149].

**Scheme 4:**
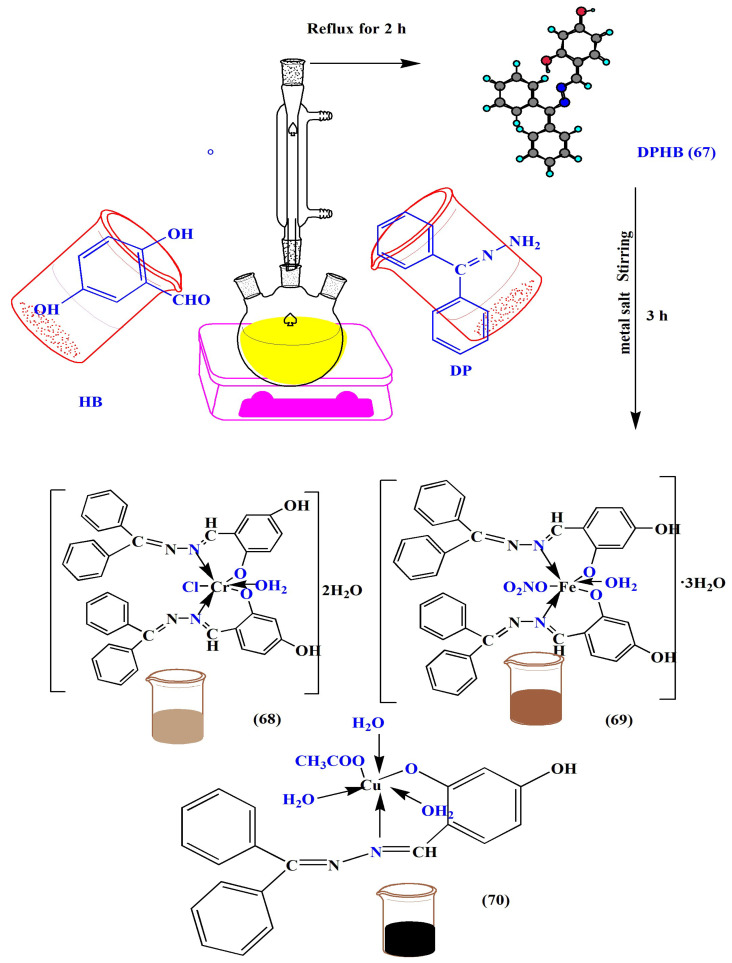
The pathway scheme of synthesis of the investigated DPHB hydrazone ligand (67) and its metal chelates (68–70). Reproduced from the permission of ref. [149].

Abu-Dief et al. (2022) prepared Fe(III), Cr(III), and Cu(II) chelates (68–70) with high yields through the reaction of aryl hydrazone ligand (DPHB) (67) with metal ions as shown in Scheme 4. Additionally, the new metal chelates have been tested anti-pathogenically and instituted to be considerably effective and associated with the equivalent DPHB ligand. The anti-proliferative performance of the tested molecules was also estimated at various positions of cancer cells and displayed vital cytotoxic performance. In addition, explanations of antioxidant performance propose that antioxidant performance comparative to usual vitamin C was verified in the molecule. As shown in Figure 8 [149].

**Scheme 5:**
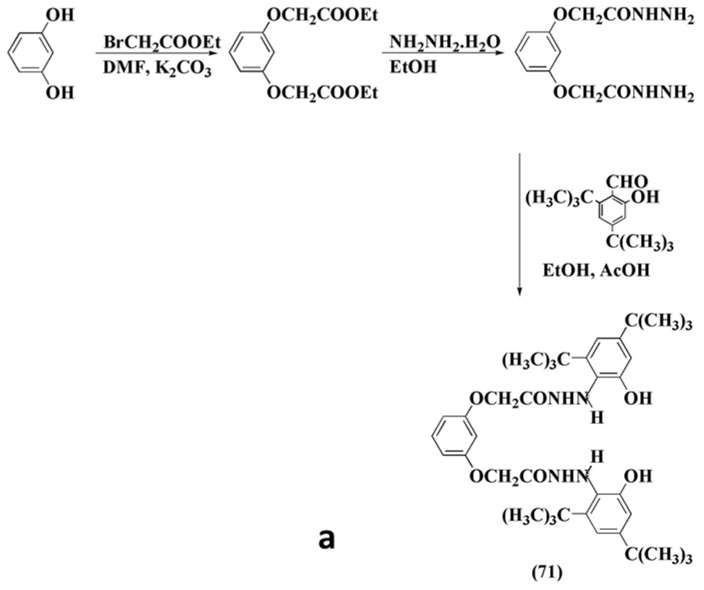

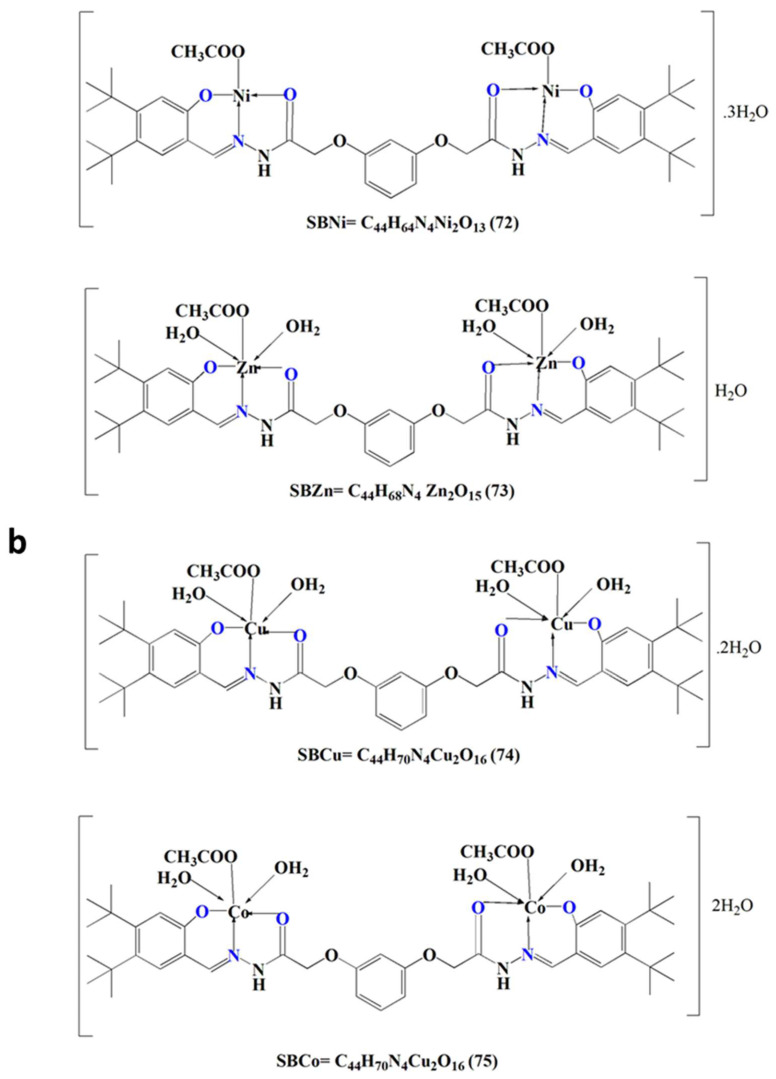
(**a**). Multi-steps for the synthesis of bis-hydrazones (SB) (71) ligand and (**b**) the structures of the prepared bis-hydrazone Schiff base complexes (72–75). Reproduced from the permission of ref. [150].

**Figure 9:**
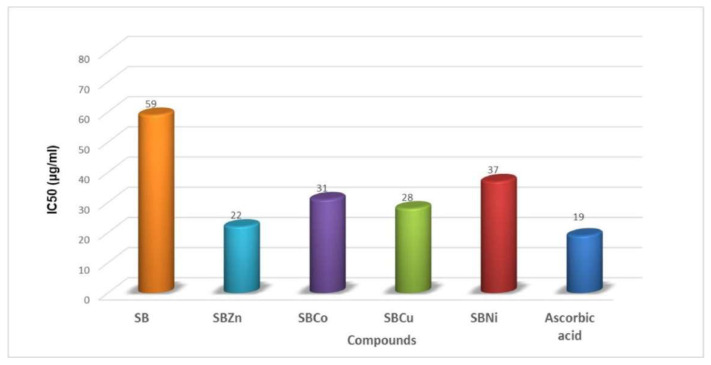
The suppression of the DPPH radical for tested compounds. Reproduced from the permission of ref. [150].

Qasem et al. (2022) prepared a new series of bis-hydrazone chelates from (N′E,N‴E)-2,2-(1,3-phenylenebis(oxy))bis(N′-(4,5-di-tert-butyl-2-hydroxybenzylidene)acetohydrazide) ligand (SB) (71) with Co^2+^, Cu^2+^, Zn^2+^ and Ni^2+^ ions (72–75), as shown in Scheme 5. Furthermore, the MTT test was applied to display the newly tested compounds towards a variety of cell lines. The antioxidant performance of the studied compounds in DMSO was estimated through the DPPH technique. These values show that the SB ligand and its metal complexes have a higher antioxidant performance, and the efficiency % of inhibitions for the tested compounds is represented in Figure 9. This decision was further corroborated by the fact that the tested chelates had similar antioxidant performance to the DPPH free radical with the reference Vitamin C. The in silico data display the low performance of the free ligand that enhanced the chelation with the Cu(II), in contrast with the practical data [150].

Sen et al. (2022) reported various requests for metal-based phenalenyl chelates, such as biological implications for metal-PLY (metal = Mn^3+^, Co^2+^, Fe^3+^, Al^3+^, and Ni^2+^) processes that are still to be identified (Figure S2). Metal-PLY (76–80) chelates were found to have acceptable antioxidant capabilities in the DPPH scavenging method. The scavenging % also improved through concentration. The Mn-PLY 2 chelate was closest to the reference ascorbic acid between the metal-PLY chelates and had the greatest antioxidant activity. For the metal-PLY chelates (Table 5), we decided that the IC_50_ data were in the demand of ascorbic acid (reference) > Mn-PLY (76) > Co-PLY (80) > Fe PLY (78) > Ni-PLY (79). Mn-PLY (76) demonstrated better antioxidant activity than other metal-PLY chelates, which could be due to the Mn^3+^/Mn^2+^ redox potential. Otherwise, ligands for the Mn-PLY (76) chelate might require improving the electron donation capability for the studied compound [151].

Parcheta et al. (2021) used several studies and the extensive literature statistics to indicate that ligand antioxidant abilities complexes with metals could have a considerable impact on radicals’ complex formation. Agreeing with their primary ideas with clarity, this effectiveness is enriched mainly with metals with strong ion potential, e.g., Cr^3+^, Fe^3+^, Ln^3+^, and Y^3+^. Chelates with molecular orbitals’ electrical charge are more effective antioxidants. The obtained measurements of antioxidant activities, such as DPPH and ferric reduced ability potential test (FRAP), were related to thermodynamic factors calculated using analytical modeling. Using experimental data obtained, the pathways of free radical formation were characterized. The HOMO energy transfer in benzoic acid derivatives changed as the number of OH groups increased. Flavonoids’ antioxidant capabilities were highly influenced by the OH group location as well as the catechol group. The number of OCH_3_ groups in phenolic acid molecules affected antioxidant performance. The use of radiation methods in the electrical structure investigation of antioxidants was planned [152].

**Figure 10:**
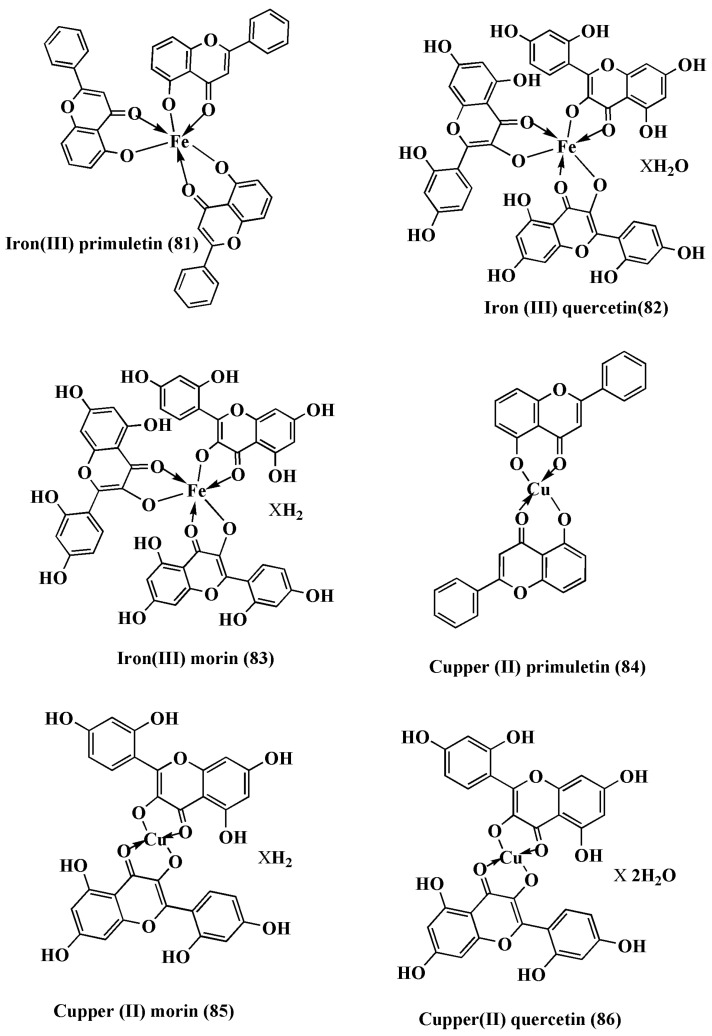
Structures of Fe(III) and Cu(II) complexes (81–86) with primuletin, quercetin and morin [153].

Mucha et al. (2021) evaluated a wide range of plant substances and their coordination compounds for their antioxidative, anti-inflammatory, anticancer, and other therapeutic effects. Because of their structural differences, flavonoids, chromones, and coumarins, as well as their coordination compounds (81–86), have diverse bioactivities (Figure 10). As well as providing an overview of the most studied antioxidant effects of such compounds, this review covers both endogenous and exogenous forms of ROS and NOS, oxidative stress-mediated lipids and peptide degradation, and the therapeutic effects of antioxidant defense systems, including plant-derived antioxidants [153].

Turan et al. (2021) studied the antioxidant and enzymatic inhibition properties of a new drug ligand as well as its metallic chelates. The new chemical ligand (((E)-6-tert-butyl 3-ethyl 2-(2-hydroxybenzylideneamino)-4,5-dihydrothieno [2,3-c]pyridine-3,6(7H)-dicarboxylate) [TBHPC] (87) was generated by combining 6-tert-butyl 3-ethyl 2-amino-4,5-dihydrothieno [2,3-c]pyridine-3,6(7H)-dicarboxylate with 2-hydroxy benzaldehyde. The metal chelates of the unusual organic ligands Fe(II), Co(II), and Ni(II) (88–90), as shown in (Scheme S7), were produced and described. In vitro antioxidant methodology experiments indicated that the obtained ligand had more potent antioxidant properties than its metal chelates; however, it also had a smaller total antioxidant potential than common bioactive components. In vitro enzymatic-acting techniques were employed to assess the inhibitory activity possibility of the tested samples for AChE, BChE, and GST enzymatic. The chemical ligand was demonstrated to be the most specific inhibitor of AChE and BChE, with Ki values of 7.13 ± 0.84 μM and 5.75 ± 1.03 μM, correspondingly. Furthermore, the K_i_ values for the GST enzyme were 9.37 ± 1.06 μM for the Fe(II) chelate. Finally, the metallic chelates verified superior critical attractions with the AChE, BChE, and GST enzymes than for the organic ligand, as demonstrated in Table 6. This work identified a promising natural base ingredient for AChE and BChE that aims to further investigate the in vivo and safety prediction [154].

Abu Dief et al. (2021) prepared a series of novel chelates resulting from Cu^2+^, Pd^2+^, and Fe^3+^ ions interacting through CPTP (91) thiazole derivative ligand. In in vitro tests, the antioxidant performance of studied compounds was screened. All chelates revealed control than free ligand ineffective behavior, particularly the CPTPPd (92) compound (Scheme S8). MOE-docking simulation and drug-likeness resulted in directly favorable inhibitory properties of CPTPPd and CPTPCu (93) chelates, in contrast with in vitro results. The findings suggested enhanced antioxidant efficacy compared that of the tested ligand, as it improved as the level of the examined molecule raised. With regards to IC_50_, the analysis indicated that CPTPFe (94) had a powerful antioxidant ability with only an IC_50_ value of 31 g/mL, which is not far from that of ascorbic acid [155].

**Figure 11:**
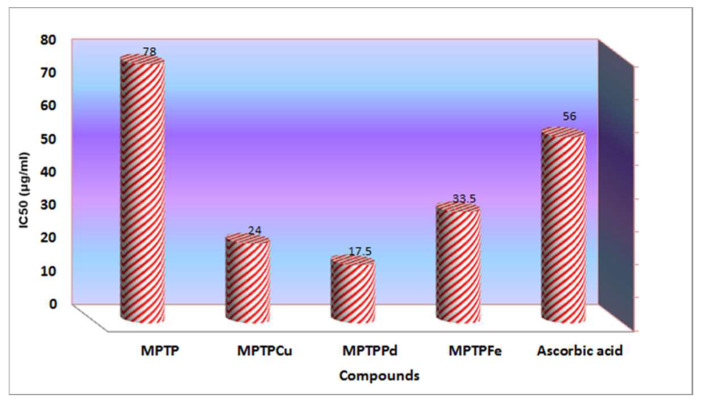
Trends in inhibition of DPPH˙ radical by the tested ligand (95) & its chelates (96–98). Reproduced from the permission of ref. [156].

**Scheme 6:**
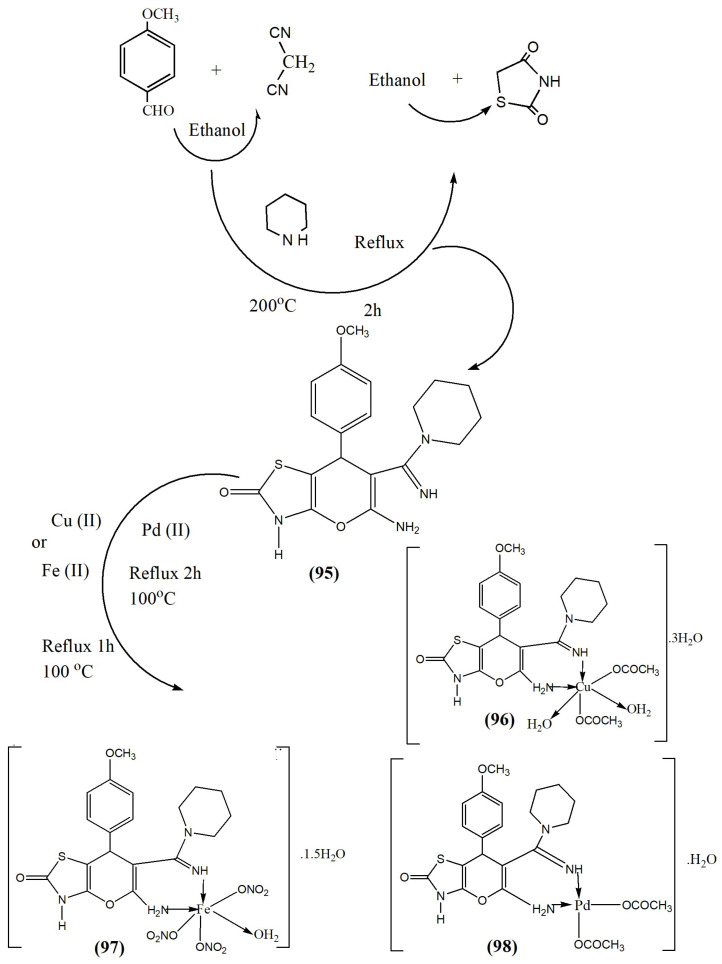
Synthetic strategy for the ligand (MPTP) (95) and its complexes, MPTPCu, MPTPFe, and MPTPPd (96–98). Reproduced from the permission of ref. [156].

Abu Dief et al. (2021) synthesized new pharmacologically active chelates from the reaction of Pd^2+^, Fe^3+^, and Cu^2+^ ions with 2-amino-6- oxo-3-(piperidinylamidino)-4 -(4methoxyphenyl)-6, 7-dihydro-pyrano[2,3-d]-5,7thiazol ligand (MPTP) (95), as shown in Scheme 6. In silico test was performed through two various methods over molecules to estimate their biological performance and grade for contact with biological structures. The MPTPPd chelate showed its importance in contact with amino acid residues and drug-like features. Antioxidant performance was studied and the chelates displayed high reactivity toward trapped free radicals (Figure 11). Such chelates (96–98) could be measured as favorable bioactive agents. The DPPH assessment presented scavenging ability for studied compounds; though, we need to evaluate the IC_50_ results to study their actual control of them. Thus, scavenging capabilities for the studied molecules were assessed for the color decay grade of DPPH [156].

**Figure 12:**
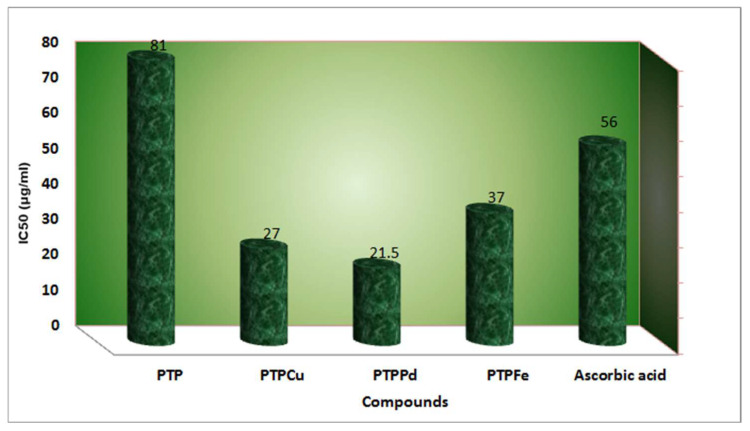
Inhibition tendency of studied PTP (99) ligand & its chelates (100–102). Reproduced from the permission of ref. [157].

**Scheme 7:**
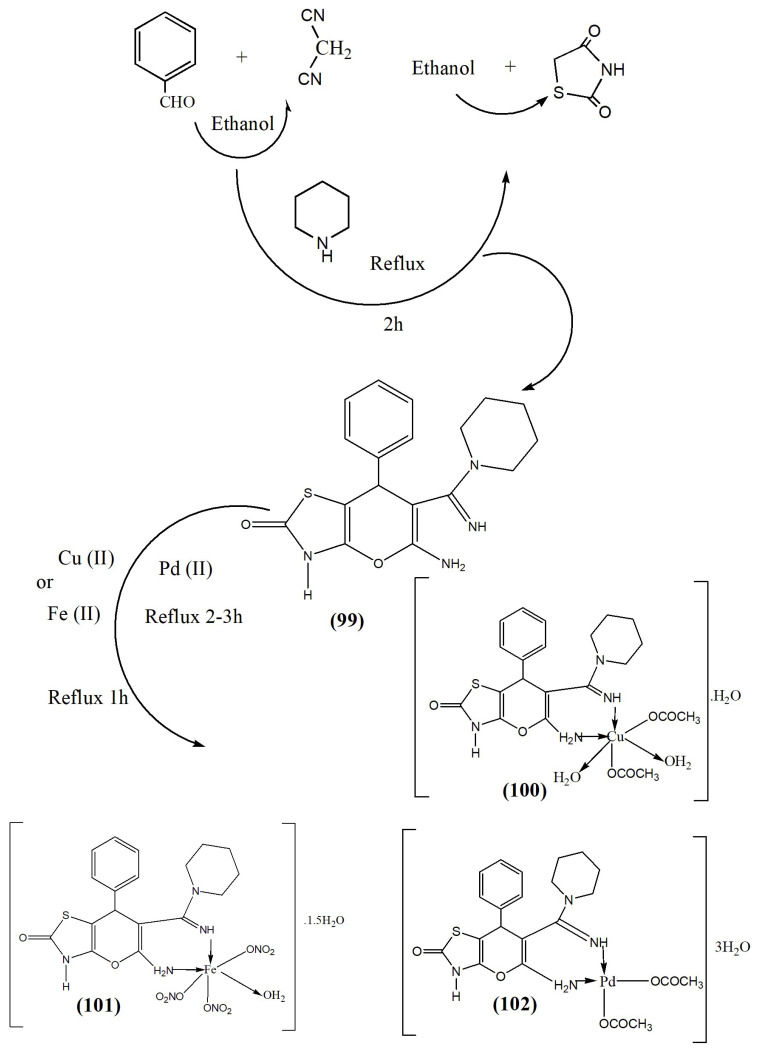
Synthetic strategy for the ligand (99) and its complexes, PTPCu, PTPFe, and PTPPd (100–102). Reproduced from the permission of ref [157].

Alzahrani et al. (2021) synthesized new bioactive chelates from the reaction of Cu^2+^, Fe^3+^, and Pd^2+^ ions through PTP (99) ligand [2-amino-6-oxo-3-(piperidinylamidino) -4-phenyl 6,7-dihydro-pyrano [2,3-d]-5,7-thiazol], as shown in Scheme 7. The ligand performed as a bidentate ligand through all metals inside (1 L:1 M) equimolar ratio. Additionally, antioxidant activity was studied and the chelates exhibited significant antioxidant performance. The tested ligand (99) and its chelates (100–102) were studied through the DPPH test, and the values pointed to vital antioxidant performance associated with standard drugs. The experiment was performed under various concentrations from the tested compound and the data were represented. As shown in Figure 12, more radical-scavenging performance of DPPH was related to minor IC_50_ data. The chelates presented a vital antioxidant performance that was greater than the standard medicine. That might be associated with the coordination structure or redox environments. Moreover, the MOE docking approach clarifies all contact properties that occurred by 2 k4 l protein, which agrees through in vitro values [157].

**Figure 13:**
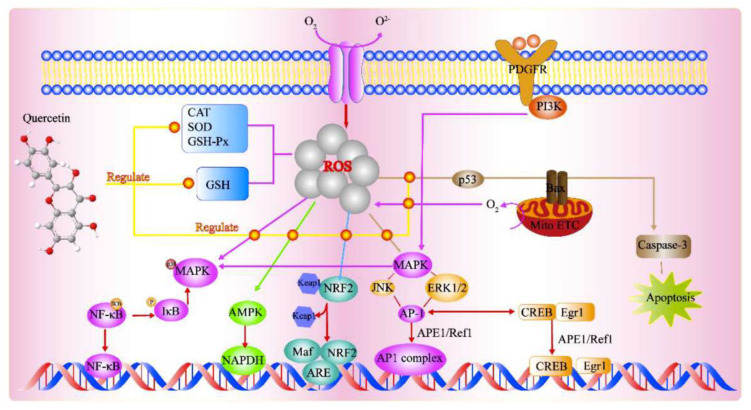
The antioxidant signaling pathway is regulated by quercetin. Reproduced from the permission of ref [158].

Xu et al. (2019) applied the tested compounds in botanic therapy and traditional Chinese therapy as a result of their effective antioxidant performance. In current years, the antioxidant performance of quercetin has been tested widely, containing its properties on glutathione (GSH), ROS, enzymatic performance, and signal transduction paths affected by green and toxicological features. Chemical tests on quercetin have mainly intensive on the antioxidant performance of its metal chelates and complex ions (Figure 13) displays the antioxidant indication paths controlled through quercetin [158].

**Figure 14:**
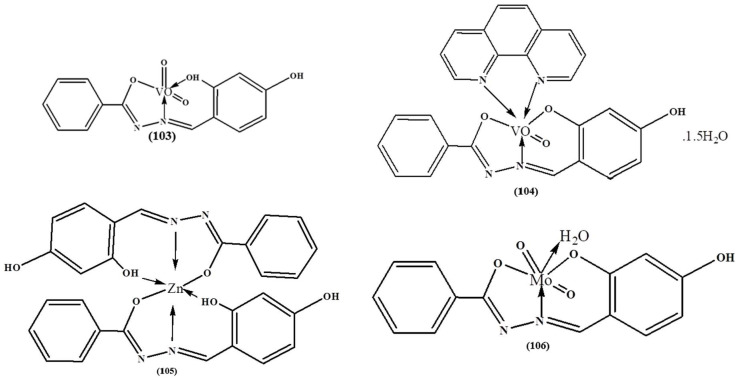

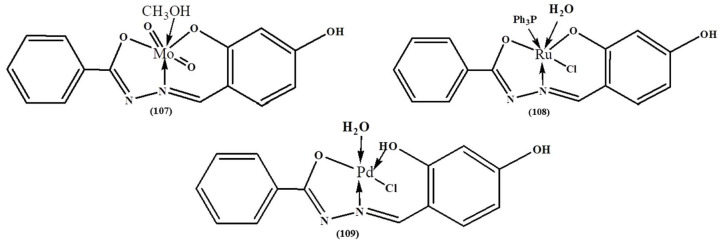
Structure of the prepared H_2_dhbh complexes (103–109). Reproduced from the permission of ref. [159].

**Figure 15:**
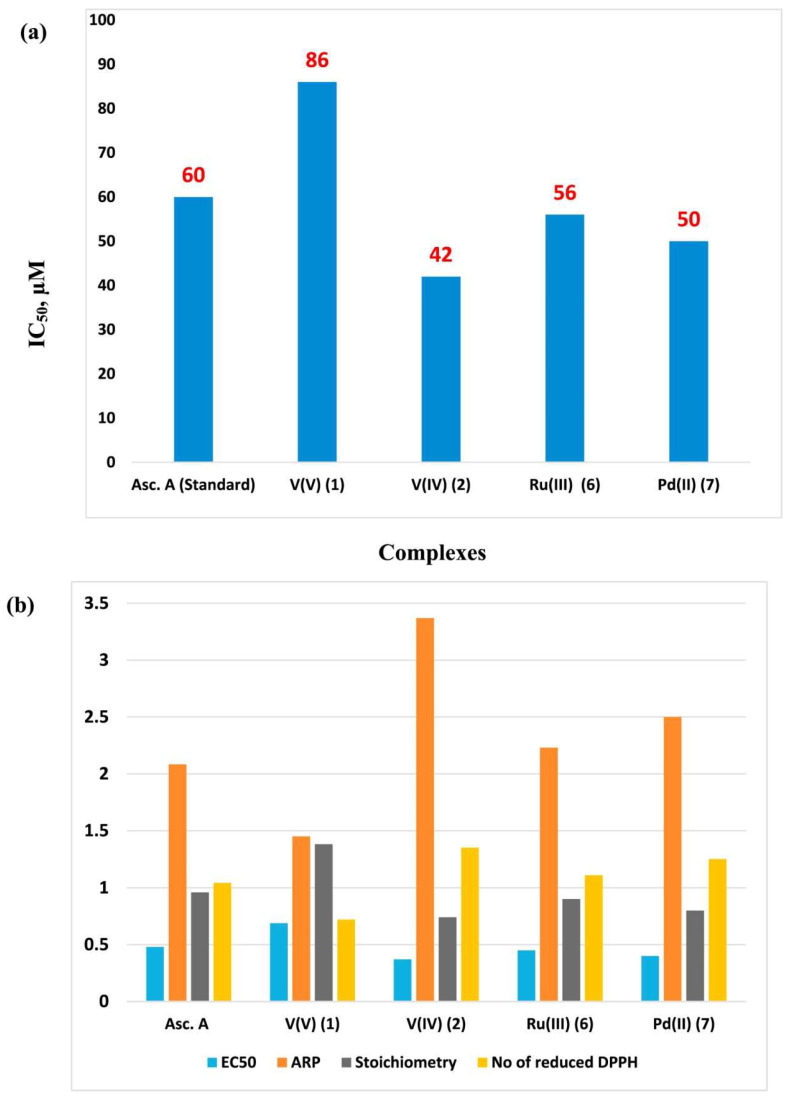
Antioxidant performance of studied compounds (103) [VO_2_(Hdhbh)], (104) [VO(phen)(dhbh)]·1.5H_2_O, (108) [Ru(PPh_3_) (dhbh)Cl (H_2_O)] and (109) [Pd(Hdhbh)Cl] estimated through DPPH radical test associated to ascorbic acid as a reference for (**a**) IC_50_ & (**b**) other antioxidant factors. Reproduced from the permission of ref. [159].

Abo afia et al. (2018) prepared and characterized new VO, Zn, Mo, Ru, and Pd chelates (Figure 14). Analytical results exhibited that H_2_dhbh ligand performed as a monobasic or dibasic tri-dentate ligand through phenolate O, azomethine N and amide O to afford [VO_2_(Hdhbh)] (103), [VO(Hdhbh)(Phen)]·1.5H_2_O (104), [Zn(Hdhbh)_2_] (105), [MO_2_(dhbh)H_2_O] (106), [MO_2_(dhbh)CH_3_OH] (107), [Ru(PPh_3_) (dhbh)Cl (H_2_O)] (108) and [Pd(Hdhbh)Cl] (109) complexes. The antioxidant performance of the tested complexes was assessed in relation to the DPPH radical, and it has been initiated that VO(IV) complex (104) demonstrated the maximum radical scavenging effectiveness equivalent to ascorbic acid as a reference antioxidant. The DNA binding assets of the tested compounds have been studied through electronic spectra with DNA cleavage through gel electrophoresis. The values presented in Figure 15 also show that V(IV) chelate (104) had a vital oxidative cleavage between other chelates [159].

## 8. Conclusions and Future Trends

The versatility of organic ligands and their derivatives’ prospect of modifying their possessions through varying the metallic chelate and the ligands open the door for the addition of other roles as well as antioxidant performance. While linking antioxidant active groups is promoting the antioxidant performance, it is still not as effective for discovering the antioxidant performance for a previously produced one, as well preparing novel antioxidant active group chelates with extra possessions. The molecular structure diversity of metal complexes has been recognized as promising for the discovery and application of new antioxidant compounds. This review covered the improvement achieved in their antioxidant activity with coordination to a wide range of metal centers and showed that various factors are involved in the antioxidant activity of a metal complex. In this context, coordination to a metal center can be seen as a useful tool to overcome the limitations of an organic antioxidant because it potentializes its activity by stabilizing the ligand structure in a more rigid geometry and because the metal complex is active by itself. For instance, the metal center acts as a modulating agent and in its high oxidation state the electron-withdrawing ability of the metal ion affects the antioxidant property of the resulting complex by transferring the electron density of the ligand to the metal center. A reverse behavior occurs when the metal is in its low oxidation state. These effects promote a considerable change in the electronic charge distribution of the ligand that facilitates the electron–proton loss and thus increases the radical scavenging ability of the resulting complex. Even while metal ions such as Mn, Fe, Cu, Co, and Zn are complex in many biological structures and play a crucial role in the functioning of organisms, it is important for reducing toxicity in the design of novel chelates antioxidants. The effective study of the sources of natural antioxidants and conniving new antioxidant molecules needs dependable systems of antioxidant performance estimation. Predictable techniques for the extent of antioxidant performance are desired and exact organizational procedures of chelates involve an extensive challenging stage. The working pH is a critical selection factor of the antioxidant assay. There seem to be examinations that are acidic (FRAP), neutral (CUPRAC), and alkaline (Folin-Ciocalteu). Furthermore, the antioxidant study’s sensitivity for both hydrophilic, as well lipophilic antioxidants, is critical. Although ABTS and CUPRAC analyses may detect hydrophilic and lipophilic antioxidants, certain systems (FRAP and Folin-Ciocalteu) solely measure hydrophilic antioxidants, as others were exclusively applicable to hydrophobic systems (DPPH). Around the same period, the color combination in the feed solution can cause absorbance changes that have a greater negative effect on the event of discoloration effects (ABTS and DPPH) than on color-formation reactions (FRAP and CUPRAC). As a result, there is still considerable potential in this study area for generating new analytical techniques for evaluating the antioxidant potential of substances, particularly in food materials. The design of biomolecule senor and their usage in the antioxidant study, for example, could be of great interest and benefit in the analysis of the project planning phase. The large variety of biorecognition elements, including enzymes, aptamers, DNA/RNA, and whole cells, are critical in designing electrical biosensors for the study of antioxidants. The accessibility, efficient analysis, and use of a small sample amount are all benefits of biosensing in the research of antioxidants using complicated data.

## Figures and Tables

**Table 1:** Antioxidant performance of (7–10) metal chelates [137].

Complex	% of Antioxidant Activity	
	DPPH	H_2_O_2_
Cd complex (7)	86.1	80.6
Cu complex (8)	78.1	71.4
Co complex (9)	74.2	72.2
Zn complex (10)	81.6	74.1
α-tocopherol	89.5	83.7

**Table 2:** DPPH radical scavenging performance of the studied compounds [141].

Tested Compound	% DRSA ± Std. Error
L1(11)	1.60 ± 0.12
L2(12)	78.45 ± 0.11
L3(13)	95.20 ± 0.14
[(benzene)Ru(L1)Cl]PF_6_ (14)	6.30 ± 0.25
[(*p*-cymene)Ru(L2)Cl]PF_6_ (15)	7.60 ± 0.23
[(benzene)Ru(L2)Cl]PF_6_ (16)	8.0 ± 0.06
[(*p*-cymene)Ru(L3)Cl]PF_6_ (17)	77.0 ± 0.02
[(benzene)Ru(L3)Cl]PF_6_ (18)	77.0 ± 0.08
[Cp*Rh(L1)Cl]PF_6_ (19)	3.70 ± 0.44
[Cp*Rh(L2)Cl]PF_6_ (20)	62.50 ± 0.12
[Cp*Rh(L3)Cl]PF_6_ (21)	81.20 ± 0.27
[Cp*Ir(L2)Cl]PF_6_ (22)	40.60 ± 0.35
[Cp*Ir(L1)Cl]PF_6_ (23)	11.0 ± 0.08
[Cp*Ir(L3)Cl]PF_6_ (24)	80.30 ± 0.11
AA	100

**Table 3:** DPPH scavenging ability (IC50, mg/mL) of the studied compounds [142].

Complex	Ligand (25)	Mn(II) Complex (26)	Ni(II) Complex (27)	Cd(II) Complex (28)	Pb(II) Complex (29)
IC_50_ values (mg/mL)	0.53 ± 0.05	0.36 ± 0.13	0.20 ± 0.11	0.43 ± 0.05	0.35 ± 0.07

**Table 4:** In vitro DPPH scavenging capacities (IC_50_, μM) of reference drugs, ligands, and their metal (II) chelates (36–55) [145].

C. No	Tested Compounds	IC_50_ (μM)
36	4-((4-(benzyloxy)-2-hydroxybenzylidene)amino)-[1,1′-biphenyl]-3-ol (BHAP)	6.13 ± 0.06
37	6-((4-(benzyloxy)-2-hydroxybenzylidene)amino)-4,6-dichloro -3-methylphenol (BHACM)	5.76 ± 0.06
38	2-((4-(benzyloxy)-2-hydroxybenzylidene)amino)-6-chloro -4-nitrophenol (BHACN)	4.98 ± 0.12
39	5-(benzyloxy)-2-(((2-hydroxyphenyl)imino)methyl)phenol (BHIMP)	7.09 ± 0.07
40	[Cupper(BHAP) (acetate)(H_2_O)_2_]	3.81 ± 0.04
41	[Cobalt(BHAP) (acetate)(H_2_O)_2_]	5.20 ± 0.15
42	[Zinc(BHAP) (acetate)(H_2_O)_2_]	4.35 ± 0.07
43	[Nickel(BHAP) (acetate)(H_2_O)_2_]	4.76 ± 0.05
44	[Cupper (BHACM) (acetate)(H_2_O)_2_]	3.23 ± 0.04
45	[Cobalt (BHACM)(acetate)(H_2_O)_2_]	5.11 ± 0.12
46	[Zinc (BHACM)(acetate)(H_2_O)_2_]	4.35 ± 0.13
47	[Nickel (BHACM) (acetate)(H_2_O)_2_]	4.66 ± 0.08
48	[Cupper (BHACN) (acetate)(H_2_O)_2_]	2.98 ± 0.09
49	[Cobalt (BHACN) (acetate)(H_2_O)_2_]	4.53 ± 0.08
50	[Zinc (BHACN) (acetate)(H_2_O)_2_]	3.76 ± 0.05
51	[Nickel (BHACN) (acetate)(H_2_O)_2_]	4.02 ± 0.06
52	[Cupper (BHIMP) (acetate)(H_2_O)_2_]	3.89 ± 0.14
53	[Cobalt (BHIMP) (acetate)(H_2_O)_2_]	5.38 ± 0.16
54	[Zinc (BHIMP) (acetate)(H_2_O)_2_]	4.22 ± 0.12
55	[Nickel (BHIMP) (acetate)(H_2_O)_2_]	4.93 ± 0.09
	Ascorbic acid	1.95 ± 0.07

**Table 5:** DPPH scavenging ability (IC_50_, mg/mL) of the reference (ascorbic acid) and the metal-PLY chelates [151].

Chelate	IC_50_ (mg/mL)
Ascorbic acid	0.042
Mn-phenalenyl complex (76)	0.059
Al-phenalenyl complex (77)	0.156
Fe-phenalenyl complex (78)	0.115
Ni-phenalenyl complex (79)	0.143
Co-phenalenyl complex (80)	0.086

**Table 6:** Ki as well as IC50 values of the organic ligand & its metal chelates [154].

	Ki (μM)			IC_50_ (μM)					
	AChE	BChE	GST	AChE	r2	BChE	r2	GST	r2
TBHPC (87)	7.13 ± 0.84 *	5.75 ± 1.03 *	11.06 ± 1.26	14.63	0.88	11.42	0.94	17.77	0.96
TBHPCFe (88)	8.89 ± 0.83	8.04 ± 1.44	9.37 ± 1.06 *	16.98	0.94	15.21	0.99	14.08	0.94
TBHPCNi (89)	9.27 ± 1.28	7.88 ± 1.86	10.87 ± 1.33	13.61	0.836	12.95	0.98	15.24	0.98
TBHPCCo (90)	7.73 ± 0.74	10.24 ± 1.66	9.98 ± 0.91	16.07	0.96	18.74	0.93	16.98	0.99
Ethacrynic acid **	-	-	0.386 ± 0.04	-	-	-	-	0.374	0.96
Tacrine ***	0.56 ± 0.06	1.32 ± 0.15	-	0.85	0.99	2.49	0.90	-	-

* The lowest Ki levels indicate the most inhibition. ** Ethacrynic acid was tested as a GST enzyme standard inhibitor. *** Tacrine served as a positive control for the AChE & BChE enzymes.

## Data Availability

Data is contained within the article or supplementary material.

## Supplementary Materials

The following supporting information can be downloaded at: https://www.mdpi.com/article/10.3390/antiox12020213/s1, Scheme S1: The structures of Schiff base ligand and their metal complexes [141]; Scheme S2: The proposed reaction pathway for formation of the metal complex with the ligand [142]; Scheme S3: For synthesis of Schiff base ligands (36–39) and their Co(II), Ni(II), Cu(II) and Zn(II) complexes (40–55) [145]; Scheme S4: Synthesis of the investigated heterocyclic metal complexes (56–59) [146]; Scheme S5: Syntheses route of compound 63 [147]; Scheme S6: Synthesis Reaction Steps of the (a) Ligand (64) and (b) Co(II) (65) and V(IV) (66) Complexes of (E)-2-(((2-((2-Hydroxyethyl)amino)quinolin-3-yl)methylene)amino) ethan-1-ol [149]; Scheme S7: Suggested structures of the ligand and its Fe(II), Co(II), and Ni(II) metal complexes; Scheme S8: Synthesis strategy of the ligand (91) and its complexes (92–94) [155]; Figure S1: New copper complexes (30–33) with terpene derivatives of ethylenediamine (34–35) [144]; Figure S2: Structures of the metal-PLY complexes [151].
