# Supplementary material for: Recent Overview of Potent Antioxidant Activity of Coordination Compounds
Source: Antioxidants (Basel). 2023 Jan 17;12(2):213. doi: 10.3390/antiox12020213 (PMC9952845; doi:10.3390/antiox12020213)
Supplement: Supplementary file 1 [file antioxidants-12-00213-s001.zip › antioxidants-2113456-supplementary.pdf]

Recent overview on potent antioxidant activity of coordination compounds

Hany M. Abd El-Lateef [1,2,*], Tarek El-Dabea[2,3], Mai M. Khalaf [1,2] and Ahmed M. Abu-Dief [2,4,*]

[1]Department of Chemistry, College of Science, King Faisal University, P.O. Box 400 Al-Ahsa 31982,
Saudi Arabia; hmahmed@kfu.edu.sa(H.M.A.); mmkali@kfu.edu.sa (M.M.K.)
[2]Chemistry Department, Faculty of Science, Sohag University, Sohag-82534, Egypt; (T.E.D.) tarekeldabea@gmail.com
[3] King Salman International University, Faculty of Science, Ras Sudr, Sinai, 46612 Egypt, tarek.eldabea@Ksiu.edu.eg
[4]Chemistry Department, College of Science, Taibah University, Madinah, P.O. Box 344, Saudi Arabia; amamohammed@taibahu.edu.sa (A. M. Abu-Dief)
* Correspondence: authors: hmahmed@kfu.edu.sa; hany_shubra@science.sohag.edu.eg (H. M. Abd El-Lateef), amamohammed@taibahu.edu.sa ; ahmedabudief@science.sohag.edu.eg (A. M. Abu-Dief).

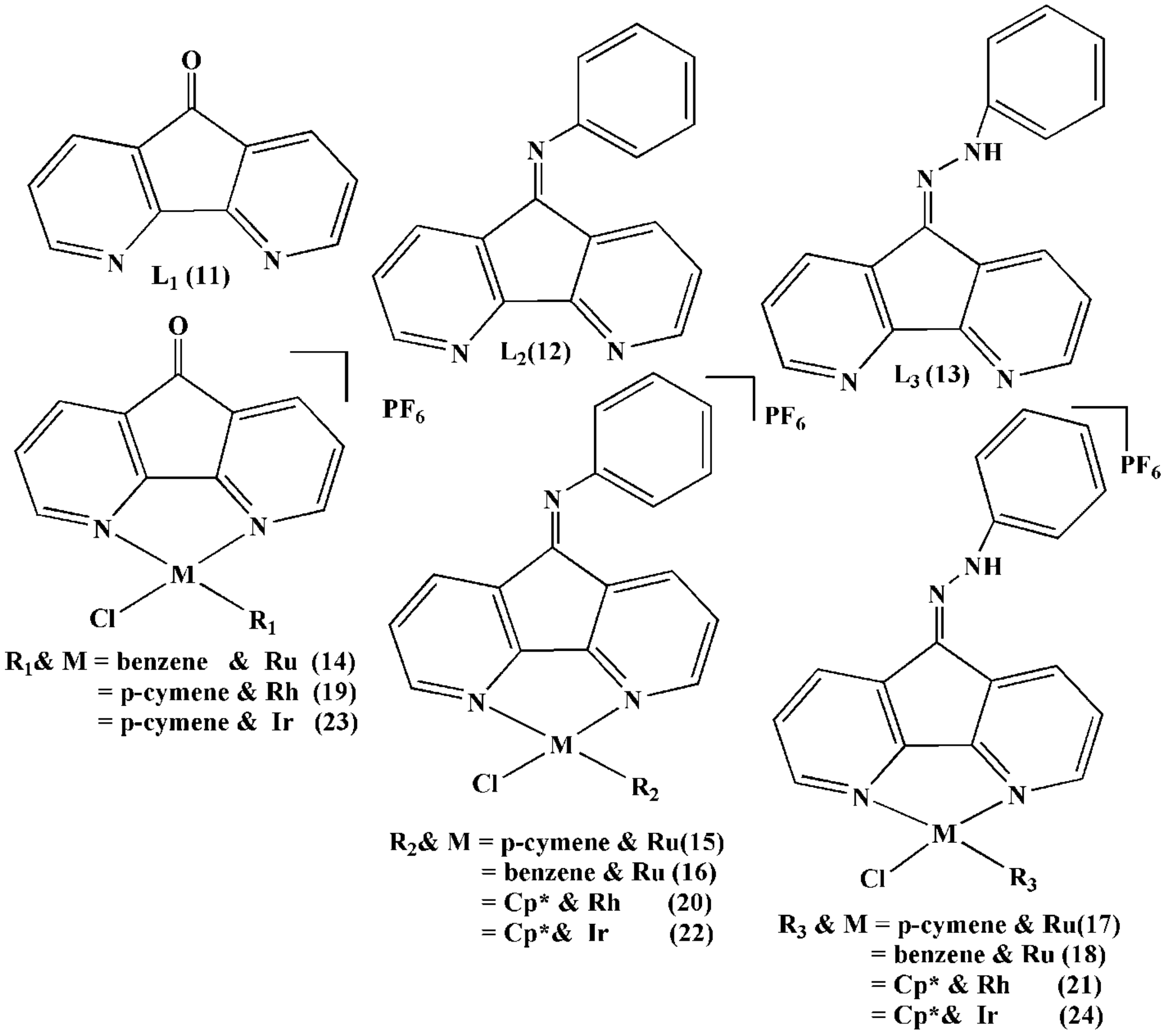

Scheme S1. The structures of Schiff base ligand and their metal complexes [141].

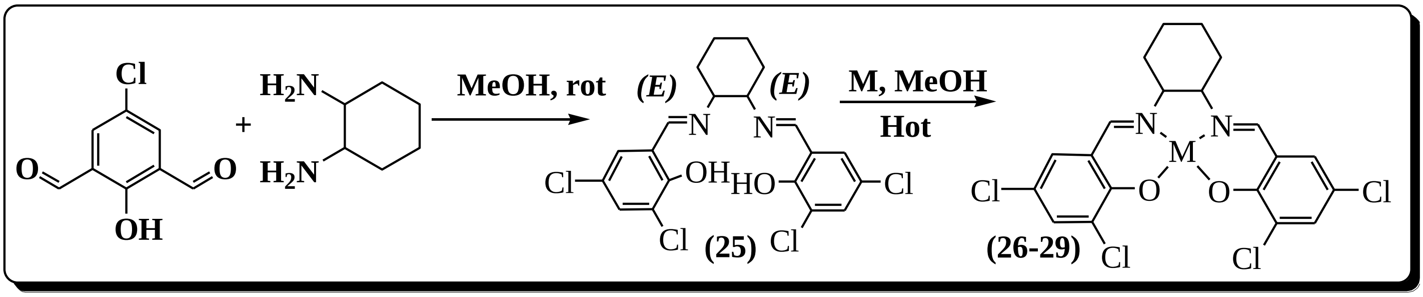

SchemeS2. The proposed reaction pathway for formation of the metal complex with the ligand [142].

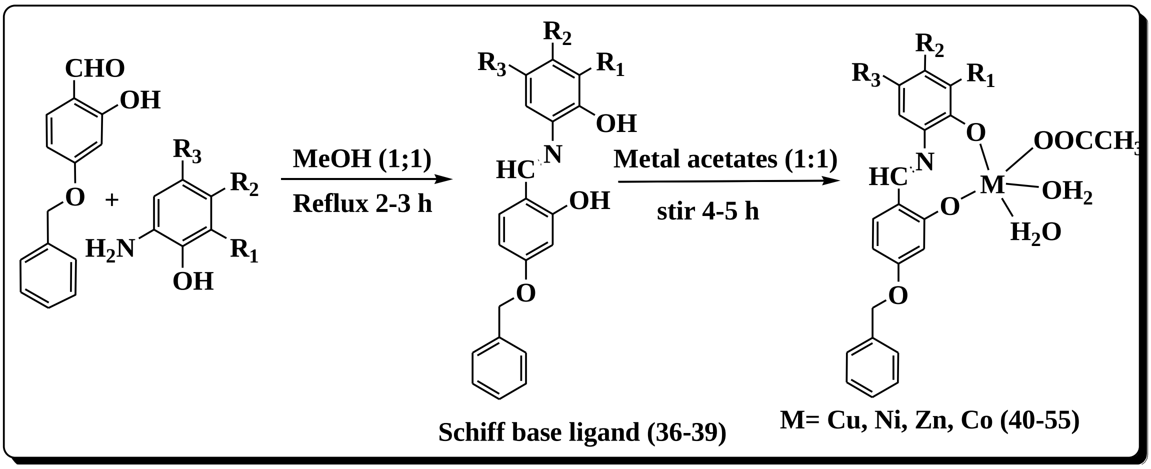

Ligands	BHAP	BHACM	BHACN	BHIMP
**R1**	H	Cl	Cl	H
**R2**	C_6H_5	CH_3	H	H
**R3**	H	Cl	NO_2	H

Scheme S3. For synthesis of Schiff base ligands (36-39) and their Co(II), Ni(II), Cu(II) and Zn(II) complexes (40-55) [145].

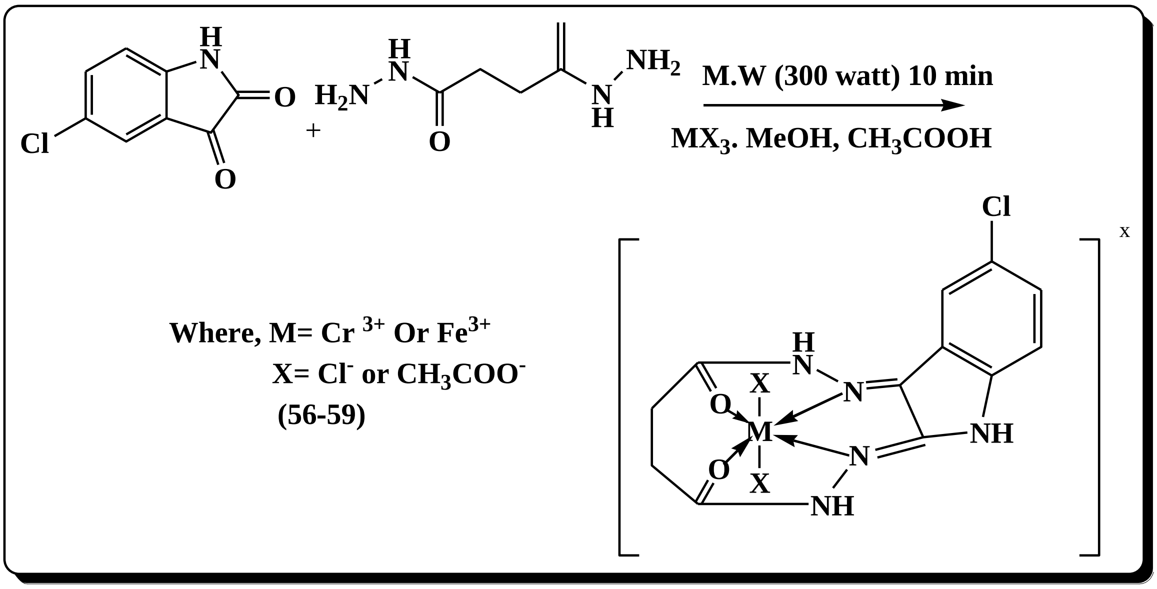

Scheme S4. Synthesis of the investigated heterocyclic metal complexes (56-59)[146].

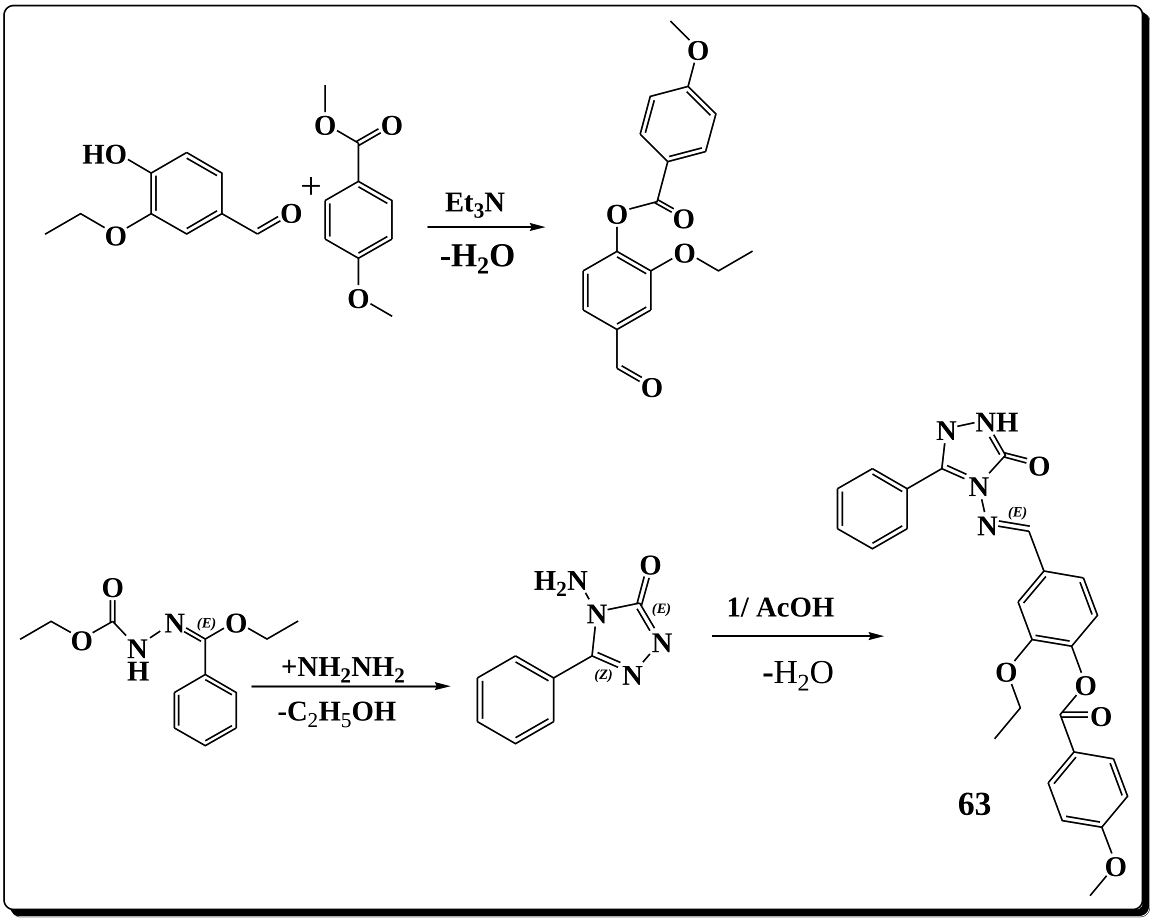

Scheme S5. Syntheses route of compound 63. [147]

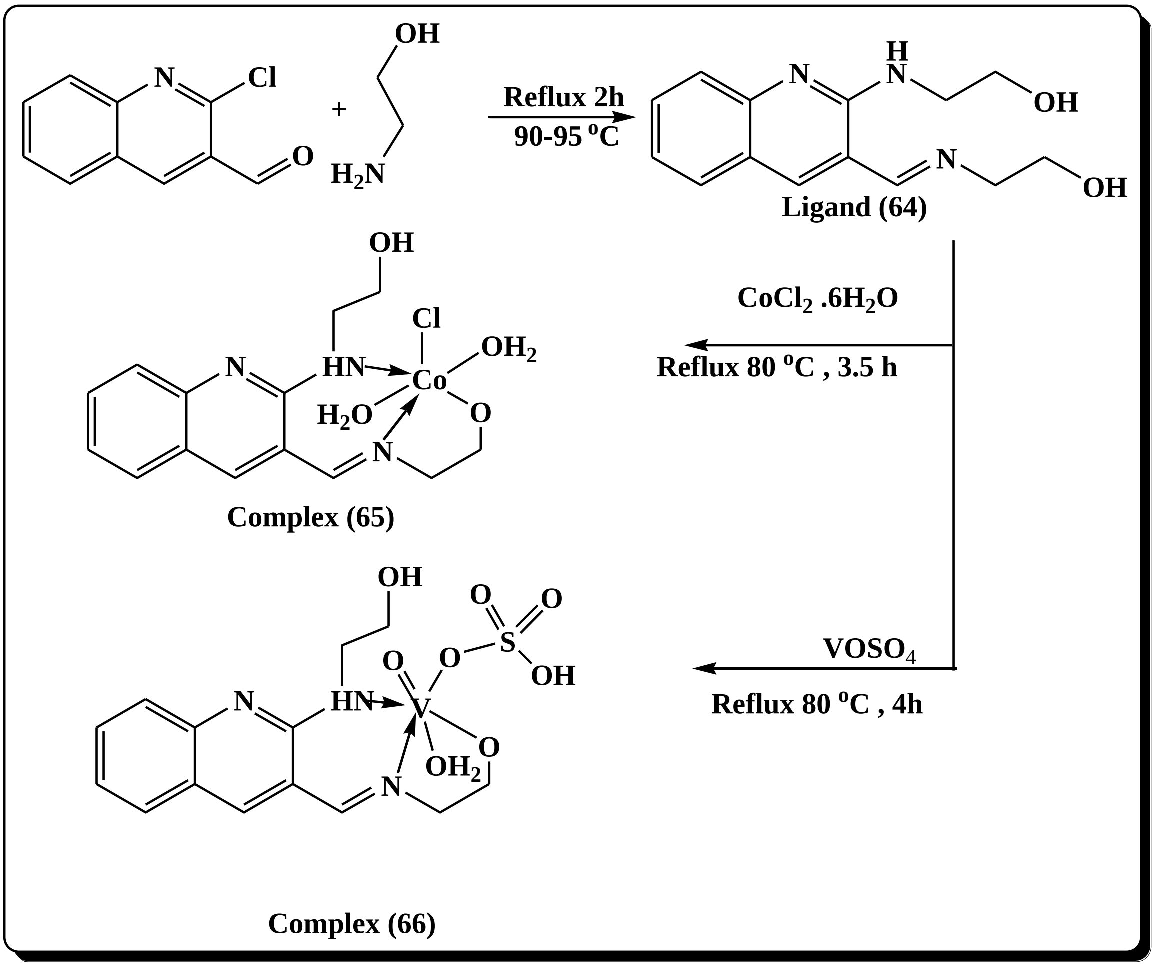

Scheme S6. Synthesis Reaction Steps of the (a) Ligand (64) and (b) Co(II) (65) and V(IV) (66) Complexes of (*E*)-2-(((2-((2-Hydroxyethyl)amino)quinolin-3-yl)methylene)amino) ethan-1-ol [149].

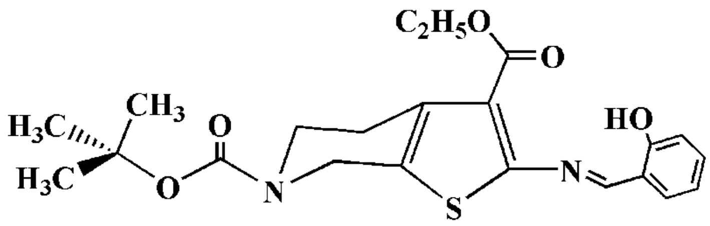

TBHPC (87)

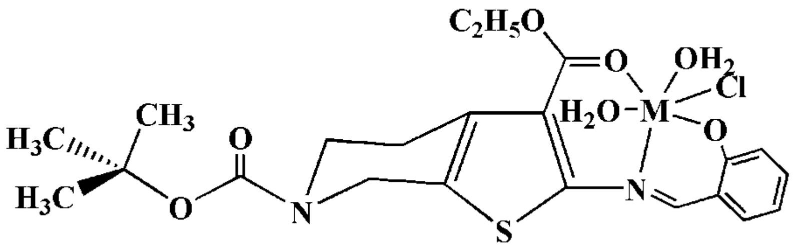

M= Fe (II), Co(II) and Ni(II) (88-90)

<u>Scheme S7 :</u> Suggested structures of the ligand and its Fe(II), Co(II), and Ni(II) metal complexes

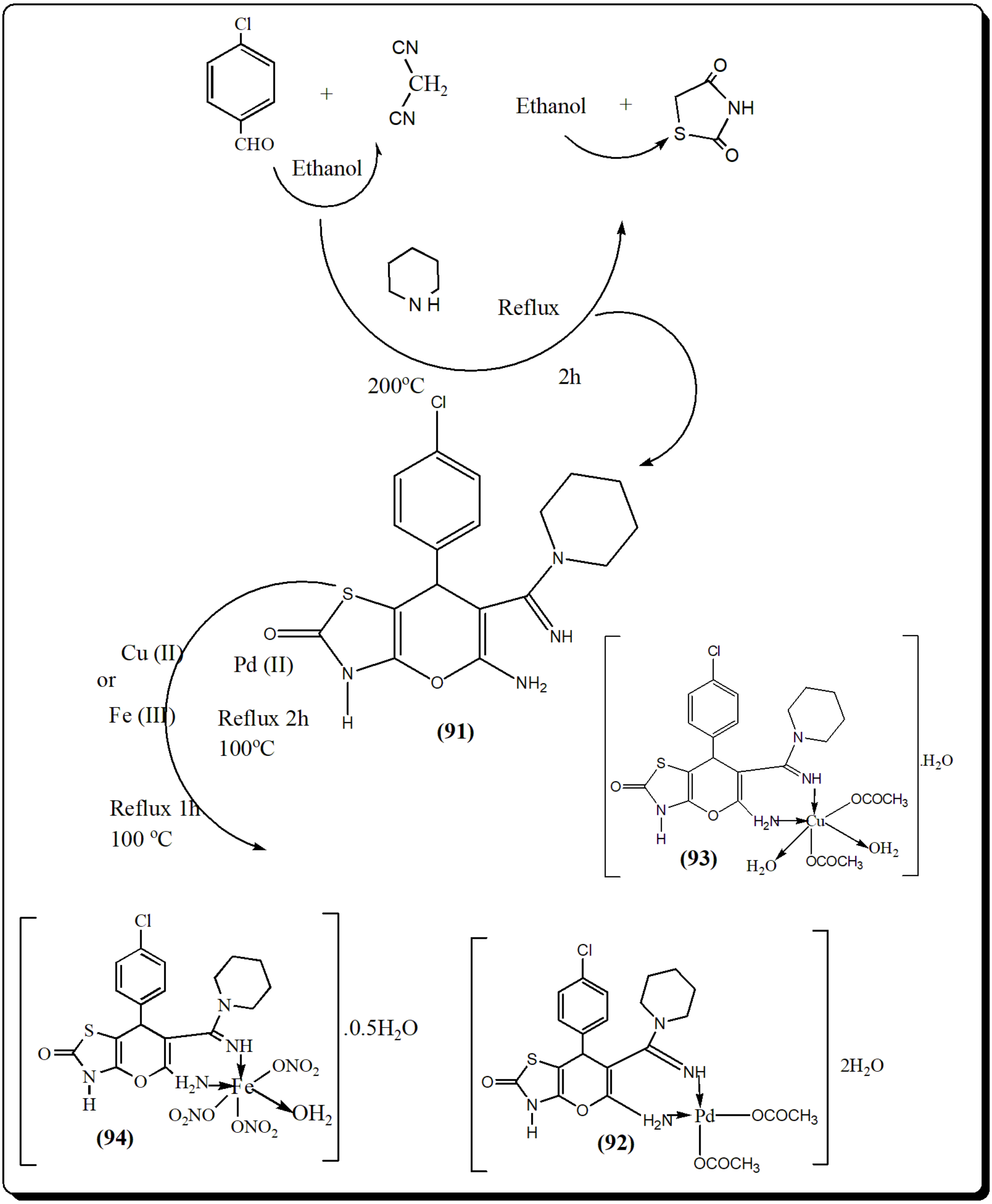

Scheme S8: Synthesis strategy of the ligand (91) and its complexes (92-94) [155]

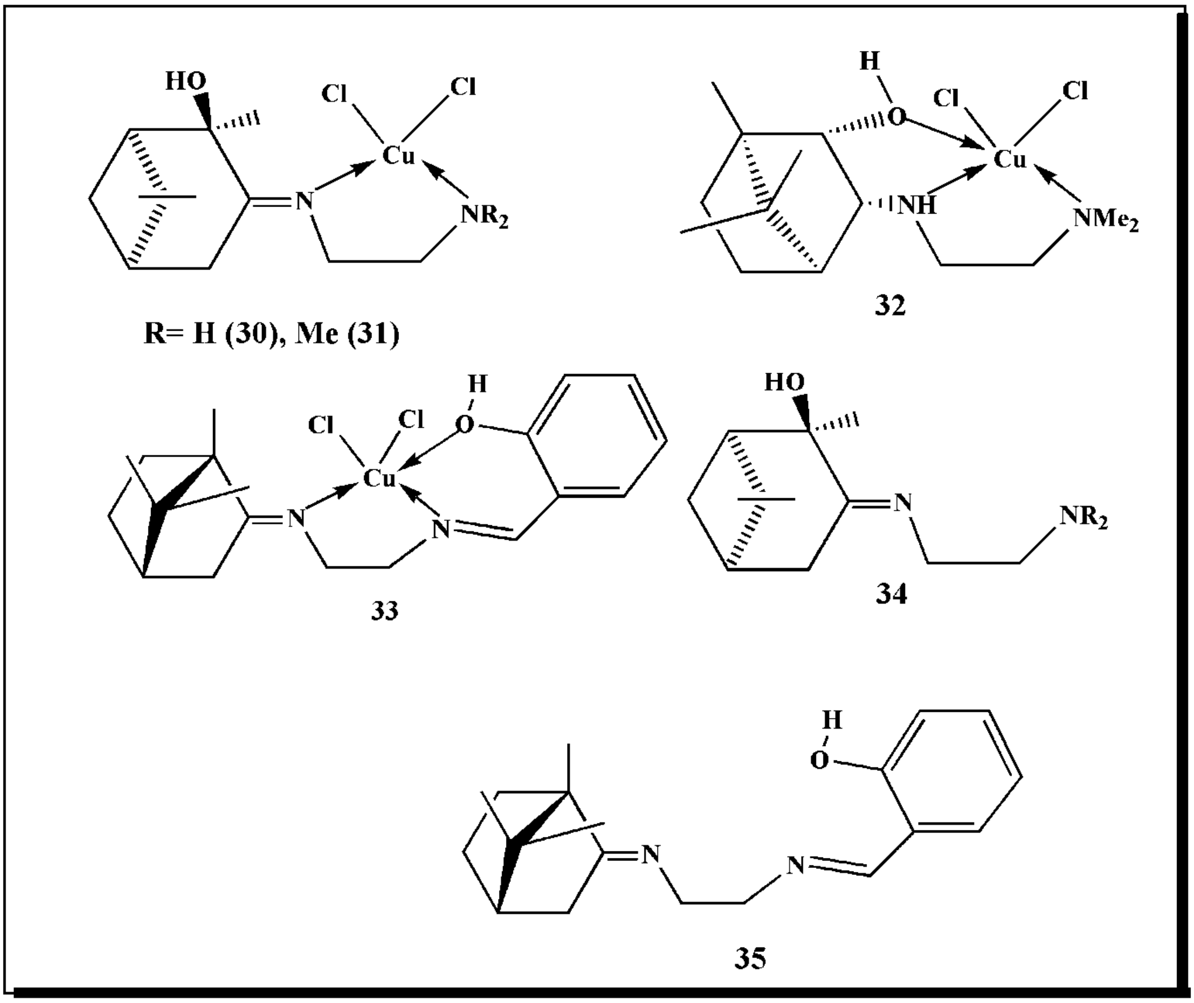

Fig. S1: New copper complexes (30-33) with terpene derivatives of ethylenediamine (34- 35) [144].

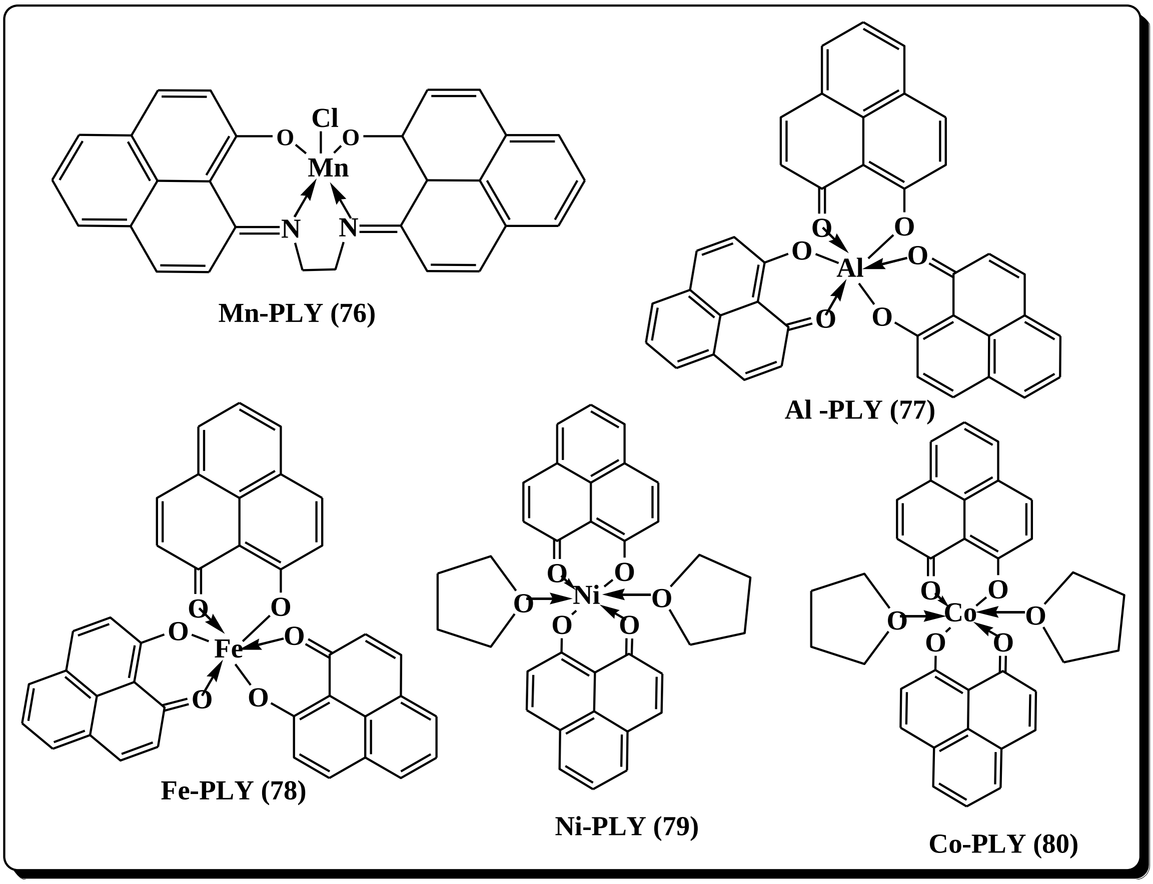

Fig. S2: Structures of the metal-PLY complexes [151].
